# Microclimate temperature effects propagate across scales in forest ecosystems

**DOI:** 10.1007/s10980-025-02054-8

**Published:** 2025-02-03

**Authors:** Kristin H. Braziunas, Werner Rammer, Pieter De Frenne, Joan Díaz-Calafat, Per-Ola Hedwall, Cornelius Senf, Dominik Thom, Florian Zellweger, Rupert Seidl

**Affiliations:** 1 Ecosystem Dynamics and Forest Management Group, School of Life Sciences, Technical University of Munich, 85354 Freising, Germany; 2 Forest & Nature Lab, Department of Environment, Faculty of Bioscience Engineering, Ghent University, 9090 Melle-Gontrode, Belgium; 3 Southern Swedish Forest Research Centre, Swedish University of Agricultural Sciences, 234 56 Alnarp, Sweden; 4 Earth Observation for Ecosystem Management, School of Life Sciences, Technical University of Munich, 85354 Freising, Germany; 5 Chair of Silviculture, Institute of Silviculture and Forest Protection, TUD Dresden University of Technology, 01737 Tharandt, Germany; 6 Swiss Federal Research Institute WSL, 8903 Birmensdorf, Switzerland; 7 Berchtesgaden National Park, 83471 Berchtesgaden, Germany; 8 Present Address: School of Environmental and Forest Sciences, University of Washington, Seattle, WA 98195 USA

**Keywords:** Climate regulation, Forest landscape model development, Microclimate, European Alps, Process-based models, Temperate mountain forests

## Abstract

**Context:**

Forest canopies shape subcanopy environments, affecting biodiversity and ecosystem processes. Empirical forest microclimate studies are often restricted to local scales and short-term effects, but forest dynamics unfold at landscape scales and over long time periods.

**Objectives:**

We developed the first explicit and dynamic implementation of microclimate temperature buffering in a forest landscape model and investigated effects on simulated forest dynamics and outcomes.

**Methods:**

We adapted the individual-based forest landscape and disturbance model iLand to use microclimate temperature for three processes [decomposition, bark beetle (*Ips typographus* L.) development, and tree seedling establishment]. We simulated forest dynamics with or without microclimate temperature buffering in a temperate European mountain landscape under historical climate and disturbance conditions.

**Results:**

Temperature buffering effects propagated from local to landscape scales. After 1,000 simulation years, average total carbon and cumulative net ecosystem productivity were 2% and 21% higher, respectively, and tree species composition differed in simulations including versus excluding microclimate buffering. When microclimate buffering was included, Norway spruce (*Picea abies* (L.) Karst.) increased by 9% and European beech (*Fagus sylvatica* L.) decreased by 12% in mean basal area share. Some effects were amplified across scales, such as a mean 16% decrease in local-scale bark beetle development rates resulting in a mean 45% decrease in landscape-scale bark beetle-caused mortality.

**Conclusions:**

Microclimate effects on forests scaled nonlinearly from stand to landscape and days to millennia, underlining the utility of complex simulation models for dynamic upscaling in space and time. Microclimate temperature buffering can alter forest dynamics at landscape scales.

**Supplementary Information:**

The online version contains supplementary material available at 10.1007/s10980-025-02054-8.

## Introduction

Forest canopies shape local environments, creating microclimatic conditions that affect ecosystem structure and processes (Geiger 1950). For example, forests modify subcanopy radiation, air and soil temperature, precipitation, wind, relative humidity, soil moisture, and snowpack duration and distribution (Chen et al. 1999; Storck et al. 2002). Near-surface climate affects a wide range of forest processes and services, including tree seedling establishment, understory species composition and cover, wildlife habitat and metabolism, decomposition rates, and disturbance intensity and effects (Chen et al. 1999; Hoecker et al. 2020; Zellweger et al. 2020; De Frenne et al. 2021; Reiner et al. 2021).

A key characteristic of forest microclimates is that temperature extremes are reduced below canopies compared to free-air conditions outside forests, leading to a microclimate buffering effect (De Frenne et al. 2021). Temperature buffering is well documented globally across multiple forest types, on average cooling maximum air temperatures by 2.7°C and warming minimum air temperatures by 1.2°C in temperate forests (De Frenne et al. 2019). Among other factors, microclimate buffering is shaped by topography and canopy structure and composition via their effects on local radiation regimes, evapotranspiration levels, and air mixing (Chen et al. 1999; De Frenne et al. 2021). Microclimate buffering by forests is expected to become increasingly important given ongoing global climate warming because of its sensitivity to macroclimate (i.e., free-air climate in open areas) temperature, with greater canopy-mediated cooling at higher maximum temperatures (De Frenne et al. 2019; Thom et al. 2020; De Lombaerde et al. 2022). As a result, microclimate warming may lag behind macroclimate warming, with implications for future forest biodiversity, species microrefugia and distributional range shifts, and carbon mitigation potential (Lenoir et al. 2017; Zellweger et al. 2020; Pastore et al. 2022; Sanczuk et al. 2023).

Forest dynamics play out at landscape scales (i.e., 10^3^ to 10^5^ ha) over long time periods, but empirical studies on how microclimate temperature affects forest processes are often restricted to local scales of observation (i.e., typically 10^-4^ to 10^0^ ha) and short-term effects. Consequently, there is an inherent scale mismatch of five to six orders of magnitude between the scale of observation and that of ecological interest. Inferring landscape-scale changes from static, plot-scale measurements is challenging, because nonlinear scaling relationships and cross-scale interactions can amplify or dampen effects (Wiens 1989; Peters et al. 2007). Furthermore, forest canopies can be highly diverse across landscapes, resulting in substantial heterogeneity in forest microclimate (Vanwalleghem and Meentemeyer 2009; Vandewiele et al. 2023). Some processes, such as disturbance and recovery, require explicit consideration of spatial patterns (Turner 2010), and disturbances in turn can alter microclimate temperature buffering (Thom et al. 2020; Wolf et al. 2021). Management decisions must also consider landscape scales to explore trade-offs among ecosystem services (e.g., Díaz-Yáñez et al. 2021), account for spatial context when altering species composition and structure (e.g., Mina et al. 2022), and mitigate climate or disturbance impacts on local communities (e.g., Jenerette et al. 2022). Understanding whether and how microclimate buffering at local scales contributes to long-term, broad-scale forest landscape change is therefore critically important for anticipating and managing future forests.

Forest landscape models are ideally suited for addressing this knowledge gap because they simulate landscape patterns as emergent outcomes of ecological processes and interactions occurring at finer spatial grains (DeAngelis and Yurek 2017). Process-based models enable projections of future forest change under no-analog conditions (Gustafson 2013), and improving climate driver representation will make projections more robust. Explicitly accounting for fine-scale microclimate temperature buffering effects could alter landscape scale outcomes, for example by modifying tree regeneration (Dobrowski et al. 2015), leading to longer term shifts in species dominance. Yet, to date microclimate temperature has not been explicitly considered in forest landscape models.

Here we developed a dynamic and computationally efficient microclimate module that incorporates microclimate temperature buffering in the individual-based forest landscape and disturbance model iLand (Seidl et al. 2012a; Rammer et al. 2024). We included microclimate temperature effects on three key forest processes that occur in the understory or near the forest floor, are dependent on temperature, and are simulated explicitly in the current version of iLand. These processes included decomposition of deadwood, litter, and soil organic matter pools; bark beetle development; and tree seedling establishment (i.e., successful first-year germination and survival).

We then used this novel microclimate module to ask, *How does accounting for microclimate temperature buffering affect forest processes from local to landscape scales?* We investigated this question in an illustrative temperate mountain forest landscape covering a broad elevational gradient (Berchtesgaden National Park, Germany). Specifically, we simulated forest and disturbance dynamics under historical climate for 1,000 years, using either daily macroclimate or microclimate temperature as the driver of the three focal subcanopy processes. We then analyzed hypothesized effects on indicators of forest dynamics at three spatial scales (local, meso, and landscape; Table 1). At the local scale (1 ha), we expected cooler microclimate temperatures under dense forest canopies to decrease decomposition (H1a) and bark beetle development rates (H1b) but maintain similar tree regeneration densities (H1c) because increases in cold-preferring species can offset decreases in warm-preferring species. At mesoscales (1-100s of ha), we expected microclimate simulations to enhance effects of disturbance mortality and associated reductions in canopy density on forest processes (H2a-c). Disturbances increase light availability in both microclimate and macroclimate simulations but additionally reduce temperature buffering in microclimate simulations only. We further expected microclimate effects to vary across the elevation ranges of tree species, with the greatest differences at lower or upper range edges relative to median elevations (H3). At the landscape scale (8,645 ha), we expected increased net ecosystem productivity (NEP; H4a) and total carbon (C) storage (H4b) in microclimate versus macroclimate simulations due to decreased decomposition and reduced bark beetle outbreaks (H4c) resulting from slowed beetle development. However, we expected similar forest composition (H4d) because temperature filters are likely less important for determining species occurrence compared to light and seed availability (Table 1).

**Table 1:** Spatial and temporal scales used to analyze effects of microclimate temperature buffering, analysis description, forest process and associated indicator, and hypotheses for whether microclimate simulations (“Micro”) would have lower (<), higher (>), or similar (~) values compared to macroclimate (“Macro”) simulations

Spatial scale	Temporal scale (yrs)	Description	Process	Indicator	Expected effect on process
Local (1 ha)	30	Annual average within dense forested stands (overstory LAI > 4)	Decomposition	Heterotrophic respiration	Micro < Macro (H1a)
			Bark beetle development	Completed beetle generations	Micro < Macro (H1b)
			Tree establishment	Tree regeneration density (stems < 4m height)	Micro ~ Macro (H1c)
Meso (1-10s of ha)	15	Average post- minus pre-disturbance indicator values in disturbance patches (5-15 years since disturbance). Patches represent 10 years of cumulative wind and bark beetle disturbances.	Decomposition	Heterotrophic respiration	Micro > Macro (H2a)
			Bark beetle development	Completed beetle generations	Micro > Macro (H2b)
			Tree establishment	Tree regeneration density	Micro > Macro (H2c)
Meso (100s of ha)	30	Relative difference in regeneration along species-specific elevation ranges [100 m bands centered on the lower bound, median, and upper bound of its elevational regeneration distribution]	Tree establishment	Tree regeneration density for six species	|Difference| at lower or upper bound > |Difference| at median of elevational regeneration distribution (H3)
Landscape (8645 ha)	1000	Average across entire forested landscape (cumulative or averaged over last 30 years)	Decomposition	Net Ecosystem Productivity	Micro > Macro (H4a)
			Decomposition	Total carbon	Micro > Macro (H4b)
			Bark beetle development	Bark beetle disturbance mortality	Micro < Macro (H4c)
			Tree establishment	Tree species composition (basal area share for trees > 4m height)	Micro ~ Macro (H4d)

## Materials and methods

### Study area

**Fig. 1:**
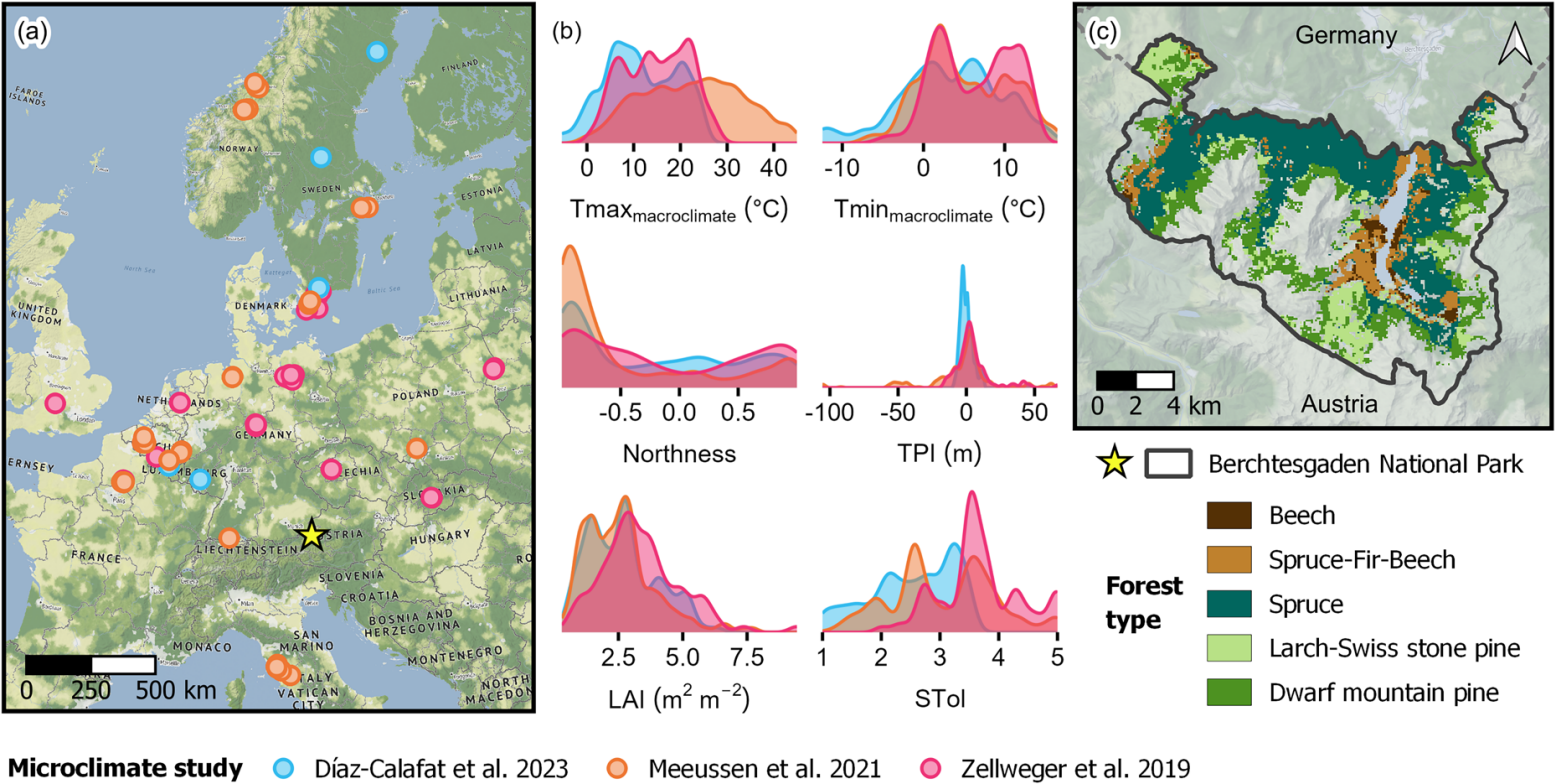
(a) Location of plots (*n* = 497, circles) across Europe from three studies where *in situ* microclimate data were collected in coniferous and broadleaved forests. Data were used here to fit empirical temperature offset models. The location of Berchtesgaden National Park is indicated by a star. (b) Density plots showing the distribution of predictor variables (see Table 2 for descriptions) across the three studies. (c) Forest simulation landscape, which includes all forested areas in Berchtesgaden National Park in Germany, and contemporary forest types. Map credits © Natural Earth, OpenMapTiles, OpenStreetMap, QGIS, Stadia Maps, Stamen Design. Beech: *Fagus sylvatica*, Spruce: *Picea abies*, Fir: *Abies alba*, Larch: *Larix decidua*, Swiss stone pine: *Pinus cembra*, Dwarf mountain pine: *Pinus mugo*

Berchtesgaden National Park is a 20,808 ha topographically complex, temperate landscape (44% of which is forested) ranging from 603-2,713 m in elevation in the northern front range of the European Alps (Figure 1). The climate is cool and wet, mean annual temperature decreases (from 7 to -2 °C) and annual precipitation increases (from 1500 to 2800 mm) with elevation, and precipitation is highest during summer. Lower elevation, submontane to montane forests are dominated by European beech (*Fagus sylvatica* L.); mixed stands of Norway spruce (*Picea abies* (L.) Karst.), silver fir (*Abies alba* Mill.), and beech; or relatively homogeneous and widespread stands of Norway spruce due to historical legacies of timber harvest and replanting. Higher elevation, subalpine forests transition from spruce-dominated to European larch (*Larix decidua* L.), Swiss stone pine (*Pinus cembra* L.), and shrubby patches of dwarf mountain pine (*Pinus mugo* Turra) near the upper treeline (~1,750 m). Dominant forest disturbance agents include European spruce bark beetles (*Ips typographus* L.) and wind, although patch sizes and annual area disturbed tend to be small relative to total forested area (< 1 ha median patch size and < 0.3% annual area disturbed between 1986 and 2020; Senf et al. 2017; Maroschek et al. 2023). Following its establishment in 1978, management ceased in a core zone covering 75% of the park. In the remainder, management activities are restricted to ungulate management, bark beetle mitigation, forest restoration, and cattle grazing in non-forested areas.

### Simulation model

The process-based model iLand simulates forest development and landscape change as an emergent outcome of species-specific, individual tree responses to abiotic drivers, disturbances, management, and competition for light (Seidl et al. 2012a; Seidl and Rammer 2024; Rammer et al. 2024). Forest processes such as productivity and biomass allocation, intrinsic and disturbance-related mortality, seed production and dispersal, and tree establishment are modeled from basic ecological principles (*sensu* Gustafson 2013). Seedlings and saplings are simulated as regeneration cohorts until reaching 4 m in height, when they are recruited as individual trees. Tree crowns shade their neighbors and the subcanopy environment, modifying microclimate light availability (2 m horizontal resolution), but until this study microclimate temperature buffering effects had not yet been incorporated. Carbon is tracked in live, dead, and soil pools, with photosynthesis, respiration, disturbance, management, and decomposition affecting fluxes among pools and to the atmosphere. Spatially explicit disturbance modules include abiotic disturbances such as wind (Seidl et al. 2014) and biotic disturbances such as bark beetles (Seidl and Rammer 2017). Bark beetle disturbances consider the life cycle of the beetle, climate-driven outbreak initiation and interactions with windthrow, spatially explicit spread, species identity and size of potential host trees, and stress-related susceptibility to colonization. Detailed model documentation is available at https://iland-model.org (Seidl and Rammer 2024).

### Empirical temperature offset models

We fit linear mixed effects models (LMMs) predicting microclimate temperature offset (°C) using data from 497 widely distributed field plots in European coniferous and broadleaved forests from three studies (Zellweger et al. 2019; Meeussen et al. 2021; Díaz-Calafat et al. 2023; Figure 1; Table S1). In each field plot, daily minimum and maximum microclimate temperature were measured at ~1m height for one to two years between 2017 and 2021, and macroclimate temperature was either acquired from a nearby weather station or measured in nearby open areas with no canopy cover (generally a nearby grassland site). Microclimate temperature offsets were calculated as microclimate minus macroclimate temperature (Equations S1-S2), meaning negative values represent cooler forest understory temperatures. Temperature data were previously reviewed and cleaned in each study, and we performed additional quality checks to identify snow days (i.e., when the microclimate sensor was covered in snow), erroneous time periods, and extreme outliers. To further reduce outlier effects and improve data normality while maintaining seasonal variation, we calculated the monthly average of daily minimum and maximum temperature offsets (hereafter, “average daily”). Separate LMMs were then fit to predict average daily minimum and maximum temperature offsets (*n* = 7,755 observations). Predictors included macroclimate temperature, topography [northness, topographic position index (TPI)], and forest structure and composition [overstory leaf area index (LAI), overstory shade tolerance as a proxy for species composition and differences in canopy architecture] as fixed effects and study (*n* = 3) as a random intercept effect to account for methodological or other differences among studies not captured by the fixed effects (Figure 1b, Table 2, Table S1). Predictors, including study, were not highly correlated (all squared scaled generalized variance inflation factors < 1.6; Fox 2016; see Supporting Information for additional detail).

**Table 2:** Variables used in average daily microclimate temperature offset models

Variable	Short name	Units	Description
Fixed effects			
Average daily minimum macroclimate temperature	Tmin_macroclimate_	°C	Monthly average of daily minimum free-air temperature; only used in predicting minimum offset
Average daily maximum macroclimate temperature	Tmax_macroclimate_	°C	Monthly average of daily maximum free-air temperature; only used in predicting maximum offset
Northness	Northness	dim[-1,1]	Cosine of topographic aspect
Topographic position index	TPI	m	Relative topographic position calculated as plot elevation minus mean elevation within a 500m radius
Leaf area index	LAI	m^2^ m^-2^	Projected leaf area per unit area (one-sided), calculated as the sum of foliage biomass times specific leaf area across all individual trees. Updated annually in iLand.
Shade tolerance	STol	dim[1,5]	Weighted mean shade tolerance across tree species, weighted by relative basal area. 1=very light-demanding, 5=very shade tolerant. Updated annually in iLand.
Random intercept effect			
Study	Study	3 levels	Categorical variable, name of the study associated with each microclimate dataset (see Figure 1)

### Microclimate module and effects on forest processes

We predicted average daily minimum and maximum microclimate temperature offset in iLand at 10m spatial resolution using the empirically derived temperature offset models and dynamically derived predictor variables from iLand (Table 2). Predictors were truncated to the maximum and minimum values used in model fitting to avoid extrapolating beyond the range of values used to train the models. We averaged minimum and maximum offset to derive average daily mean microclimate temperature offset. Temperature offsets were updated monthly for each 10m cell but were added to daily macroclimate temperature to match the time step of iLand, meaning microclimate temperature varied daily in the simulation model.

To evaluate simulated microclimate buffering in iLand, we compared seasonal variability, differences among forest types, and spatial patterns of temperature offsets with ecological expectations and with an independent, wall-to-wall microclimate dataset. This dataset consisted of Berchtesgaden summer temperature offset maps derived by combining *in situ* microclimate and macroclimate observations from 2021 with LiDAR-derived metrics of forest structure and topography (Vandewiele et al. 2023). Because daily downscaled (100 m) historical macroclimate data used in iLand were only available for 1980–2009 (Thom et al. 2022), evaluation simulations used contemporary forest and topographic conditions with macroclimate from a year representing average historical mean annual temperature for the landscape (5.7 °C, in 1988).

We simulated effects of microclimate temperature buffering on three temperature-dependent processes that occur in the forest understory: decomposition, bark beetle development, and tree establishment. These processes were already implemented and tested in previous versions of iLand; in the new microclimate version of the model, affected processes use daily microclimate rather than macroclimate temperature as inputs. Forest processes occurring within or near the top of the canopy, such as tree primary production, were driven by macroclimate temperature in all simulations. Macroclimate temperatures in iLand refer to free-air temperature at 2 m height and 100 m horizontal resolution, derived from interpolated historical weather station data (Thom et al. 2022). To calculate microclimate temperature in iLand, offsets were averaged at 100 m spatial resolution across stockable 10m cells (i.e., excluding areas such as rocks or water bodies that are unable to become forested), and then added to macroclimate temperature (Equations S1-S2).

Decomposition rates of snags, downed wood, litter, and soil organic matter are simulated based on first order decay kinetics in iLand. The reference decay rate is sensitive to a climate modifier that accounts for temperature and moisture (Adair et al. 2008; Seidl et al. 2012b). This modifier affects both the transition rate between carbon pools (e.g., downed wood to soil) and the rate of heterotrophic respiration to the atmosphere (Kätterer and Andrén 2001). To account for temperature buffering effects, the microclimate module calculates this modifier from mean microclimate instead of macroclimate temperature.

In Central European forests, iLand simulates the dynamics of the European spruce bark beetle *Ips typographus* (henceforth “bark beetle” for brevity). Bark beetles can produce multiple generations per year, with bark temperature influencing development rates and sister brood initiation (Baier et al. 2007). In the newly developed microclimate module, bark temperature is calculated from maximum microclimate instead of maximum macroclimate air temperature, and overwintering success is based on minimum microclimate rather than minimum macroclimate temperature (see Seidl and Rammer 2017 for the equations representing the respective processes). Other climate-sensitive aspects of bark beetle spread and outbreak intensity, such as outbreak initiation and host tree susceptibility, are driven by macroclimate temperature, summer precipitation, and drought stress.

Successful tree establishment in iLand relies on passing multiple, species-specific abiotic filters. These filters include minimum winter temperature, winter chilling requirements, and growing degree days, which act as thresholds either allowing or preventing establishment (Nitschke and Innes 2008). Other abiotic conditions, including soil water availability and growing season frost events, also modify establishment probabilities if thresholds are met (see Seidl et al. 2012b and Hansen et al. 2018 for a detailed description). In the newly developed microclimate module, abiotic filters are calculated from daily minimum (minimum winter temperature, growing season frost) or mean (winter chilling requirements, growing degree days) microclimate rather than macroclimate temperature.

### Initial conditions and simulation experiment

Contemporary forest conditions (year 2020); historical climate, soils, and topography; wind and bark beetle disturbance regimes; and tree species parameters for Berchtesgaden National Park were derived and rigorously evaluated by Thom et al. (2022) and have been used in multiple studies (Albrich et al. 2022; Dollinger et al. 2023; Braziunas et al. 2024). To assess effects of microclimate temperature buffering from local to landscape scales, we simulated 1,000 years of forest development under historical climate and disturbances, with no forest management, and starting from contemporary forest conditions including all major and most minor tree species in Berchtesgaden National Park. Simulations either used macroclimate or microclimate temperature as drivers of decomposition, bark beetle development, and tree establishment processes. We simulated 10 replicates of each condition (macroclimate or microclimate) to account for variation due to probabilistic processes in iLand (Rammer et al. 2024). To further isolate the importance of macroclimate versus microclimate temperature as the driver of forest dynamics, each replicate followed a randomly selected sequence of climate years and wind events drawn from the previously compiled historical data representing the period 1980 to 2009 (Thom et al. 2022).

### Analyses across scales

We analyzed the effect of microclimate temperature buffering on indicators of the three focal forest processes by comparing simulations driven with macroclimate or microclimate at three spatial scales and variable temporal scales (Table 1; see Supporting Information for additional detail). At local scales, we compared forest process indicators in dense forested stands. At mesoscales, we quantified disturbance effects as the post- minus pre-disturbance indicator value within disturbance patches. Also at mesoscales, differences in tree establishment along species-specific elevation ranges were assessed for a subset of representative species varying in elevational range and temperature sensitivity: beech and silver fir (submontane-montane zone, warm preferring), spruce and Swiss stone pine (subalpine, cold preferring), and sycamore maple (*Acer pseudoplatanus* L.) and larch (montane and subalpine, respectively, temperature indifferent; Ellenberg and Leuschner 2010). At the landscape scale, we compared NEP, carbon storage, disturbance mortality, and tree species composition after 1000 years of forest development (Table 1). We then compared relative differences in landscape-scale indicators between the first and last 30 simulation years and with local-scale indicators to consider how microclimate effects changed over time and across scales. Because data were generated via a simulation experiment, comparisons prioritized ecologically meaningful interpretations such as relative differences between mean indicator values and variability based on standard errors, rather than tests of statistical significance (White et al. 2014).

## Results

### Empirical temperature offset models

In order of predictor importance, buffered (i.e., warmer) minimum microclimate temperatures were associated with higher TPI, more northerly aspects, lower shade tolerance, cooler minimum macroclimate temperatures, and higher LAI (Equation S3, Table 3). Model fit for average daily minimum temperature offset was conditional R^2^_c_ = 0.24 (full model), marginal R^2^_m_ = 0.07 (fixed effects only), and root-mean-squared-error (RMSE) = 1.4 °C (Figure S6a). In order of predictor importance, buffered (i.e., cooler) maximum microclimate temperatures were associated with warmer maximum macroclimate temperatures, higher LAI, more northerly aspects, lower shade tolerance, and lower TPI (Equation S4, Table 4). Model fit for average daily maximum temperature offset was conditional R^2^_c_ = 0.47, marginal R^2^_m_ = 0.29, and RMSE = 2.7 °C (Figure S6b). Models represented seasonal variability in microclimate temperature buffering well (Figure S6c-d).

**Table 3:** Linear mixed effects model coefficients and random intercept effect standard deviation for average daily minimum temperature offset models, fit to *n* = 7,755 observations

Variable	Estimate	Standard error (fixed effects) or standard deviation (random intercept effect)	t	p
Fixed effects				
(Intercept)	1.4570	0.3877	3.7590	0.03
TPI	0.0158	0.0009	18.1540	< 2.00 × 10^-16^
Northness	0.2627	0.0237	11.0990	< 2.00 × 10^-16^
STol	-0.2031	0.0224	-9.0560	< 2.00 × 10^-16^
Tmin_macroclimate_	-0.0248	0.0029	-8.6360	< 2.00 × 10^-16^
LAI	0.0227	0.0127	1.7960	0.07
Random intercept effect				
Study	–	0.6614	–	–

*Tmin*_*macroclimate*_ Average daily minimum macroclimate temperature: *LAI* Leaf area index; *STol* Shade tolerance; *TPI* Topographic position index

**Table 4:** Linear mixed effects model coefficients and random intercept effect standard deviation for average daily maximum temperature offset models, fit to *n* = 7,755 observations

Variable	Estimate	Standard error (fixed effects) or standard deviation (random intercept effect)	t	p
Fixed effects				
(Intercept)	0.9767	0.9428	1.0360	0.37
Tmax_macroclimate_	-0.1932	0.0034	-57.3680	< 2.00 × 10^-16^
LAI	-0.3948	0.0250	-15.7920	< 2.00 × 10^-16^
Northness	-0.5729	0.0466	-12.2910	< 2.00 × 10^-16^
STol	0.4419	0.0442	9.9900	< 2.00 × 10^-16^
TPI	0.0140	0.0017	8.0790	7.51 × 10^-16^
Random intercept effect				
Study	–	1.6145	–	–

*Tmax*_*macroclimate*_ Average daily maximum macroclimate temperature, *LAI* Leaf area index, *STol* Shade tolerance, *TPI* Topographic position index

### Dynamically simulated temperature offsets in iLand

**Fig. 2:**
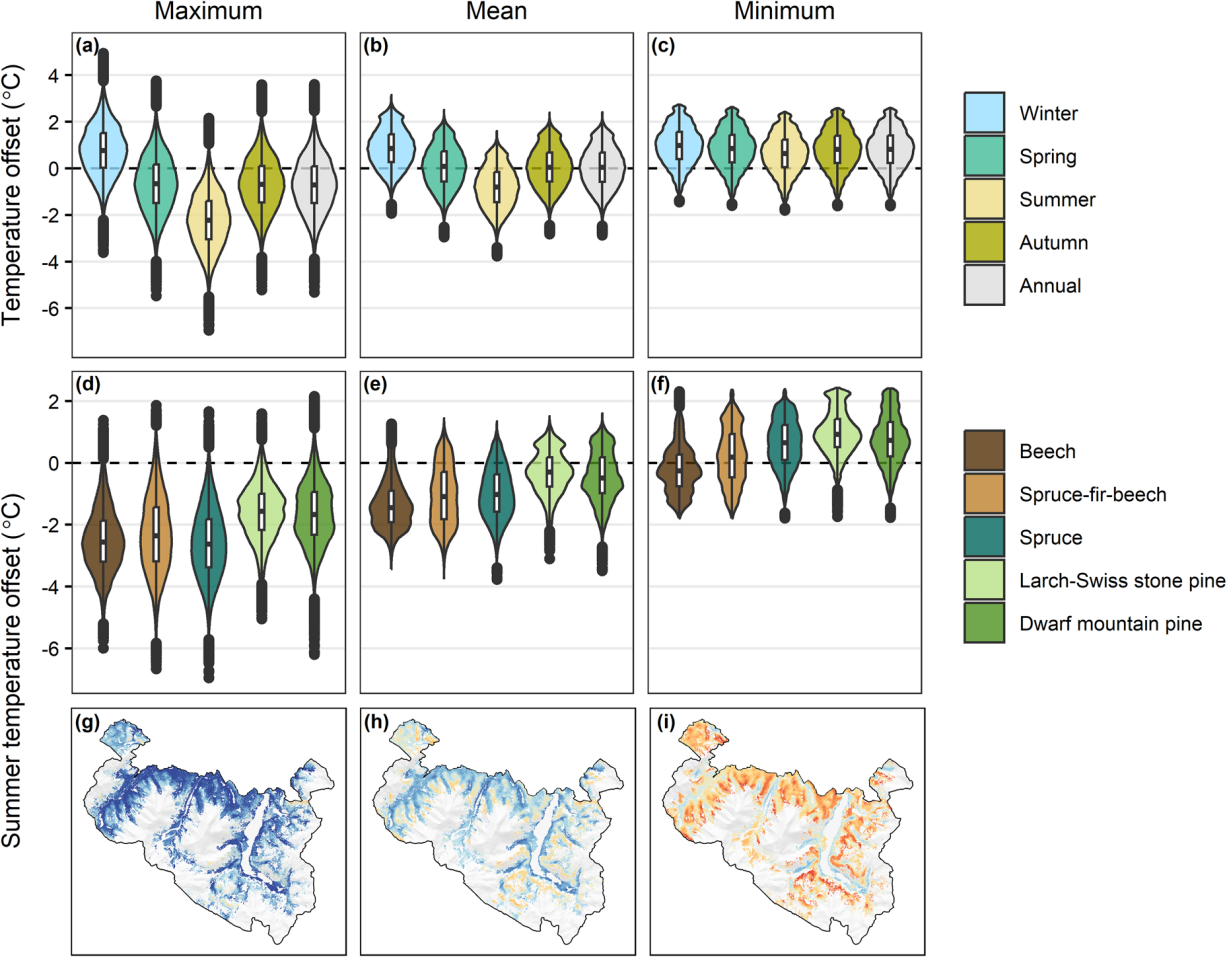
Simulated maximum, mean, and minimum temperature offsets in Berchtesgaden National Park using the newly developed microclimate module in iLand, based on contemporary forest conditions and a year with average historical climate conditions. (a-c) Seasonal and annual temperature offsets across all forested cells (864,466 observations per season at 10 m spatial resolution). (d-f) Summer (June-August) temperature offsets by forest type. (g-i) Maps of summer temperature offsets (values truncated to -3 and 3). Temperature offsets are microclimate minus macroclimate temperature. Beech: *Fagus sylvatica*, Spruce: *Picea abies*, Fir: *Abies alba*, Larch: *Larix decidua*, Swiss stone pine: *Pinus cembra*, Dwarf mountain pine: *Pinus mugo*

Daily temperature offsets averaged -0.7 °C for maximum, 0.1 °C for mean, and 0.8 °C for minimum temperatures across the entire forested landscape during a year with average historical climate conditions (mean of 864,466 observations at 10 m spatial resolution; Figure 2a-c; Table S2). Relative to maximum and mean macroclimate temperatures, forests tended to warm microclimate temperatures in the winter (average offset = 0.8 and 0.9 °C for maximum and mean, respectively) and cool microclimate temperatures in the summer (-2.2 and -0.8 °C), with spring and autumn temperature offsets falling in between these extremes. Forests consistently tended to warm minimum microclimate relative to macroclimate temperatures across the full year.

Microclimate temperature buffering differed among forest types and across the landscape, and simulated mean summer offsets during an average historical climate year (1988) aligned with independent offset maps derived from field data and LiDAR collected in 2021 (Spearman’s ρ = 0.47; Figure S8). Mean summer microclimate temperatures were cooled the most in beech-dominated forests (average offset = -1.3 °C), followed by spruce-fir-beech and spruce (both -1.0 °C), dwarf mountain pine (-0.4 °C), and larch-Swiss stone pine forest types (-0.3 °C; Figure 2e). Trends were similar for maximum and minimum temperature offsets, except that spruce forests cooled maximum temperatures slightly more than beech forests (-2.6 versus -2.5 °C for spruce and beech, respectively; Figure 2d) and warmed minimum temperatures more than spruce-fir-beech forests (0.6 versus 0.2 °C for spruce and spruce-fir-beech, respectively; Figure 2f). Lower (i.e., more negative, cooler) temperature offsets occurred at lower elevations and valley bottoms whereas higher (i.e., more positive, warmer) offsets occurred at higher elevations and exposed ridges (Figure 2g–i).

### Local-scale effects

**Fig. 3:**
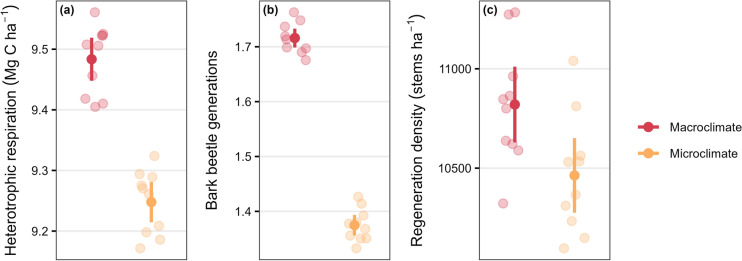
Local scale indicators of forest processes for simulations without (macroclimate, red) versus with (microclimate, yellow) temperature buffering included in the model. (a) Heterotrophic respiration as an indicator of decomposition, (b) completed bark beetle generations as an indicator of bark beetle development, and (c) regeneration density for stems < 4 m height as an indicator of tree establishment. Values are the annual average for dense forested stands (LAI > 4) over the first 30 simulation years. Solid points: mean across all replicates, error bars: two standard errors, jittered shaded points: mean value for each simulated replicate (*n* = 10 replicates)

In dense forested stands, annual heterotrophic respiration was 2% lower (9.25 vs. 9.48 Mg C ha^-1^), the number of completed bark beetle generations 20% lower (1.37 vs. 1.72 generations), and tree regeneration density 3% lower (10,463 vs. 10,820 stems ha^-1^) in microclimate compared to macroclimate simulations (Figure 3). Regeneration composition shifted in dense forested stands, with slightly higher proportions of some subalpine species and slightly lower proportions of some submontane to montane species in microclimate versus macroclimate simulations (Figure S9).

### Mesoscale effects

Variability among patches exceeded variability between macroclimate and microclimate simulations for post- minus pre-disturbance changes in heterotrophic respiration rates and tree regeneration densities (Figure S10). However, including microclimate temperature buffering more consistently enhanced post-disturbance bark beetle development (mean change 0.17 versus 0.09 generations ha^-1^ and increases in 46% versus 32% of patches in microclimate versus macroclimate simulations, respectively). Disturbance patch numbers and sizes differed between macroclimate (283 patches, 1-59 ha in size) and microclimate simulations (165 patches, 1-49 ha).

**Fig. 4:**
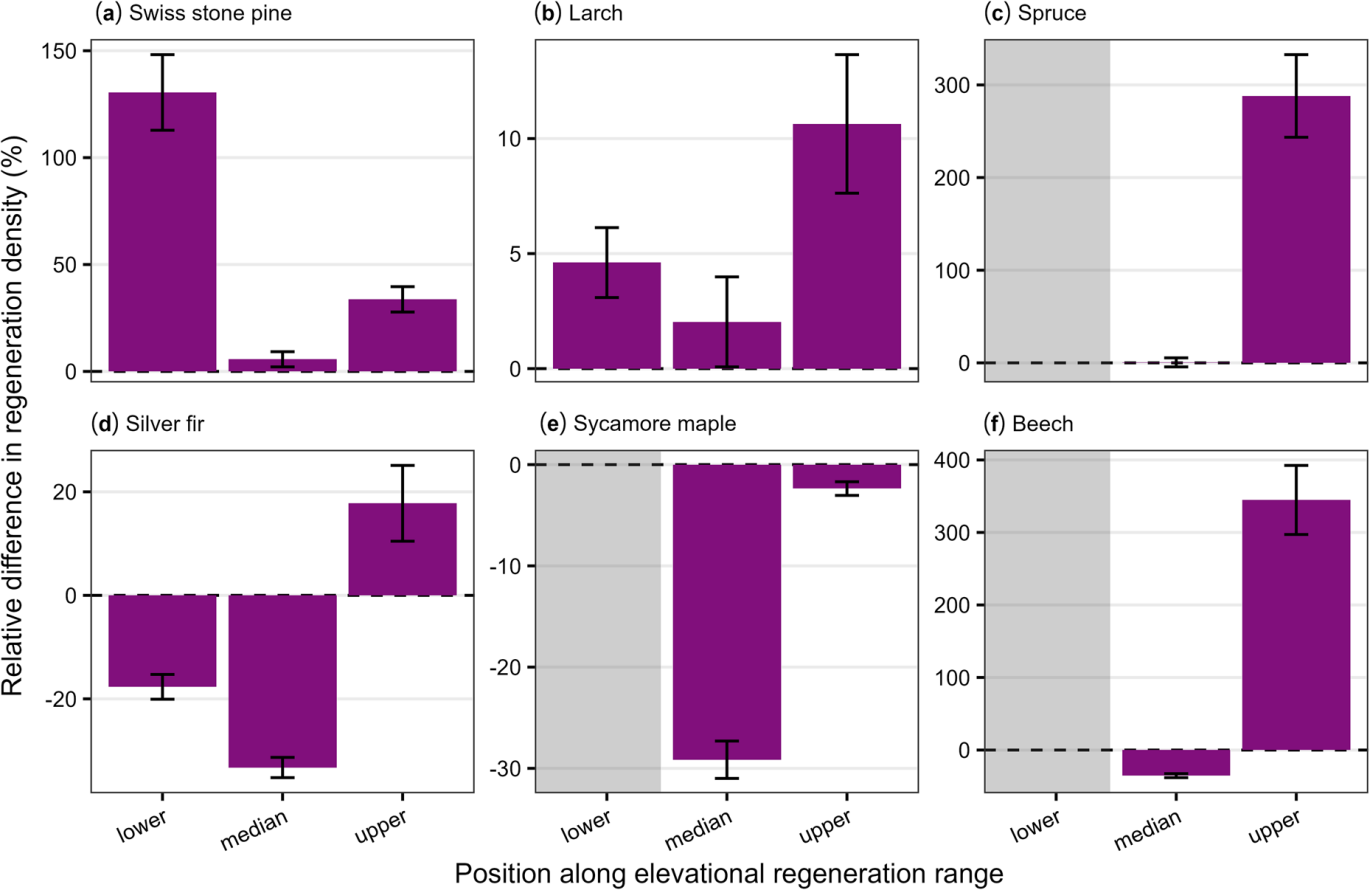
Relative difference in regeneration density for six representative tree species along their elevation ranges (100 m bands centered on the lower bound, median, and upper bound of their elevational distribution). Positive values indicate increased regeneration when microclimate temperature buffering is included in the model. Bar height: mean, error bars: two standard errors (*n* = 10 replicates), gray boxes: excluded from analysis because they fell below the minimum landscape elevation. Swiss stone pine: *Pinus cembra*, Larch: *Larix decidua*, Spruce: *Picea abies*, Silver fir: *Abies alba*, Sycamore maple: *Acer pseudoplatanus*, Beech: *Fagus sylvatica*.

Microclimate temperature-driven differences in tree regeneration varied among representative species and along their elevational ranges (Figure 4). Four of the six species (Swiss stone pine, larch, spruce, beech) responded more to microclimate effects at the lower or upper bounds relative to median values within their elevation range, and most species tended to increase in density at higher elevations. Subalpine species usually increased in regeneration density (Figure 4a–c), whereas submontane and montane species decreased at lower and median elevations (Figure 4d–f). Within these elevation zones, temperature-indifferent species (larch, sycamore maple) tended to be less sensitive than other species to microclimate effects on regeneration density.

### Landscape-scale effects

**Fig. 5:**
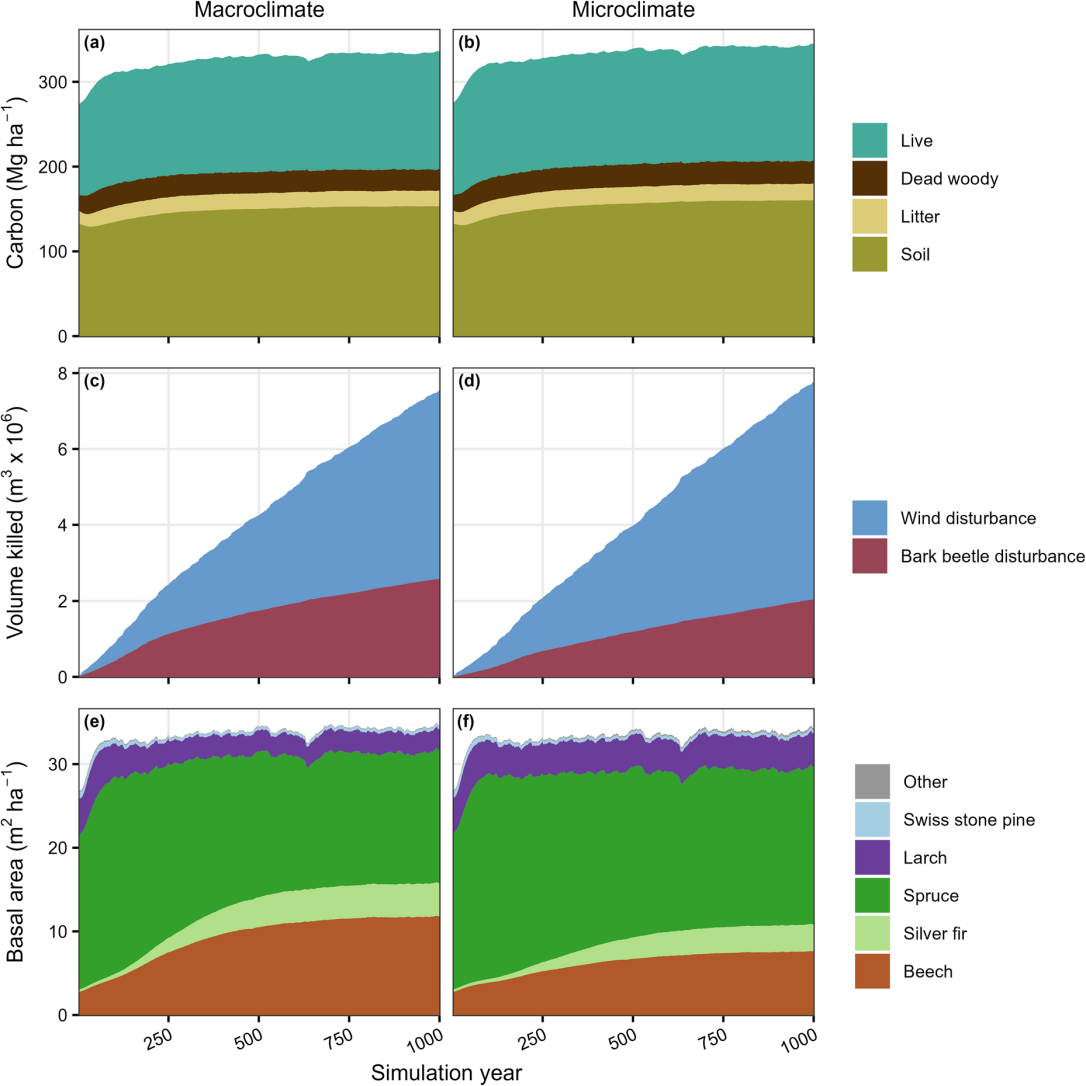
Landscape scale trajectories for (a-b) total carbon and carbon pools, (c-d) cumulative disturbance mortality due to wind and bark beetles, and (e-f) tree species basal area, without (left) or with (right) microclimate temperature buffering effects included in the model. Plots show the mean value from 10 simulated replicates. Beech: *Fagus sylvatica*, Spruce: *Picea abies*, Fir: *Abies alba*, Larch: *Larix decidua*, Swiss stone pine: *Pinus cembra*, Dwarf mountain pine: *Pinus mugo*

After 1,000 years, total carbon and cumulative NEP were higher (by 2 and 21%, respectively) and forest species composition differed in microclimate versus macroclimate simulations (Figures 5, S11-S12). Increases in total carbon were primarily driven by increased soil carbon (7.10 Mg C ha^-1^) and partially offset by decreased live carbon (-1.64 Mg C ha^-1^). When microclimate temperature buffering was included, basal area share increased for dominant subalpine species (from 7 to 12% for larch and 46 to 55% for spruce) and decreased for dominant submontane-montane species (from 12 to 9% for silver fir and 34 to 22% for beech). Cumulative bark beetle-caused tree mortality was 21% lower in microclimate versus macroclimate simulations but windthrows more than compensated for this decline, resulting in a 3% increase in total disturbance mortality (Figure 5, S11-S12).

Relative differences between microclimate and macroclimate simulations tended to be lower in magnitude for landscape versus local-scale indicators of decomposition and tree establishment (Figure S13). However, relative decreases in bark beetle-caused mortality at the landscape scale were of greater magnitude (-45%) than decreases in bark beetle development rates at the local scale (-16%). Landscape scale differences between microclimate and macroclimate simulations increased over time for total carbon and species basal area, but not for annual NEP or disturbance mortality (Figure S12).

## Discussion

We developed the first explicit and dynamic implementation of microclimate temperature buffering in a forest landscape simulation model and found that microclimate effects cannot be neglected for simulated forest dynamics. Local effects of buffered subcanopy temperatures scaled up nonlinearly, underlining the utility of using complex simulation models for dynamic upscaling in space and time. Spatially, microclimate effects at local scales could not simply be added up to estimate landscape scale outcomes. Temporally, microclimate effects were not static, as interacting drivers (e.g., disturbances) and cross-scale feedbacks were either amplifying or dampening. By explicitly modeling microclimate temperature buffering in a process-based forest landscape model, we provide a tool that is well suited for investigating critical ecological challenges in the 21st century.

### Simulated microclimate temperature offsets aligned with expectations

Microclimate temperature offset predictions echoed ecological expectations, and offset magnitudes were within the range of empirical observations in temperate forests (De Frenne et al. 2019). Responses to predictors were consistent with previous studies that found higher buffering with increasing canopy density (von Arx et al. 2013; Zellweger et al. 2019) and under more extreme macroclimate temperatures (De Frenne et al. 2019; Thom et al. 2020). Cooler microclimates at lower topographic positions reflected cold air pooling dynamics (Pastore et al. 2022). Seasonal trends aligned with empirical studies, finding enhanced cooling of maximum temperatures during summer and lower seasonal variability for minimum temperature buffering (Zellweger et al. 2019; Meeussen et al. 2021). However, variance explained by fixed effects was low, especially for minimum temperature offsets. Differences among the three study datasets (e.g., in instrumentation, macroclimate data source, and range of predictor values) likely contributed to poor model performance.

Simulated summer microclimate temperature offsets aligned well with independent offset maps derived from field data and LiDAR (Vandewiele et al. 2023). This independent dataset was not used to train the model, yet the relative ranking of forest types and hotspots of highest and lowest buffering capacity were similar for maximum and mean offsets. Differences between datasets were likely primarily due to different microclimate measurement height. Temperatures close to the ground (15 cm for independent data) may diverge from 1 m height measurements (as simulated in iLand) due to dense understory vegetation, differential air mixing, and closer proximity to the soil surface where radiant heat transfer occurs (Geiger 1950; Campbell and Norman 1998). Overall based on this independent data comparison, we conclude that the temperature offsets simulated in this study are robust and consistent with empirically derived expectations for microclimate temperature buffering.

### Microclimate temperature buffering mattered across scales

At local scales, decomposition and bark beetle development decreased as expected (H1a-H1b; see Table 1 for hypotheses) in dense forested stands when driven by microclimate rather than macroclimate temperature. Counter to our expectations (H1c), tree regeneration densities also tended to decrease, suggesting that cooler maximum and mean temperatures drove overall responses (e.g., by reducing the likelihood of meeting growing degree day thresholds) more than warmer minimum temperatures (e.g., by reducing growing season frost events). Shifts in tree regeneration composition imply that some species benefit more from microclimate temperature buffering than others due to species-specific traits (Dobrowski et al. 2015).

At mesoscales, high variability among disturbance patches overwhelmed differences between microclimate and macroclimate simulations for all processes except bark beetle development (H2a-c). Temperature is only one factor influencing post-disturbance dynamics, and processes may be more sensitive to other disturbance-mediated factors such as amount and arrangement of dead woody biomass (e.g., heterotrophic respiration; Harmon et al. 2011), light availability (e.g., tree seedling survival and growth; Xu et al. 2023), and biotic legacies (e.g., seed supply; Gill et al. 2022). Furthermore, if disturbance severity is low, canopy gaps are small, or residual structures remain – as is frequently the case in our study landscape – disturbance effects on temperature buffering may be less pronounced (Abd Latif and Blackburn 2010; Carlson et al. 2021). Microclimate effects on tree establishment along elevational ranges generally aligned with expectations (H3). Positive effects at higher elevations suggest most species benefited from being released from minimum temperature and frost limitations.

At the landscape scale, total carbon and cumulative NEP increased as expected (H4a-b), but forest composition shifted more substantially than expected (H4d) when driven by microclimate rather than macroclimate temperature. Compositional changes highlight the role of intact forest canopies and variable topography in creating climatic conditions that favor certain species (Dobrowski et al. 2015). Shifts in landscape scale carbon storage and cycling suggest cascading effects of microclimate-driven processes on the climate regulating function of forests (De Frenne et al. 2021; Pastore et al. 2022). In addition to removing live woody carbon, forest loss could accelerate carbon losses from soil and dead pools if decomposition rates increase with warmer free-air temperatures. Bark beetle development rates were dampened as expected (H4c) but, perhaps surprisingly, did not translate into overall reductions in disturbance mortality because increasing wind disturbances more than compensated for declining bark beetle disturbances. However, this trade-off is ecologically reasonable; the dense, homogeneous stands of large Norway spruce that dominate this landscape are susceptible to both bark beetle and wind disturbances (Stritih et al. 2021). Previous studies have found similar compensatory disturbance dynamics in forests of Central Europe (Dobor et al. 2020).

### Limitations and future directions

We only considered microclimate buffering effects on temperature in this study. However, forest canopies already influence light availability and water cycling in iLand simulations (Seidl et al. 2012a). Some processes, such as decomposition, are therefore already influenced by canopy-mediated effects on precipitation and potential evapotranspiration (Adair et al. 2008). Additionally, other climate-sensitive processes occur underneath forest canopies. For example, future model development could explore microclimate effects on surface fuel moisture and associated dynamics of fire ignition, spread, and severity (Rothermel 1983).

Our aim was to identify a generalizable, robust, dynamic, and computationally efficient approach for representing microclimate temperature effects on forest processes and landscape outcomes (i.e., to find the Medawar zone of optimal model complexity; Grimm et al. 2005). For this reason, we used a simple empirical equation to predict temperature offsets rather than a process-based approach rooted in environmental biophysics (e.g., as in microclimc; Maclean and Klinges 2021). We capitalize on the strengths of a process-based forest model such as iLand by simply substituting microclimate for macroclimate temperature for focal processes, allowing effects to propagate across spatial scales and over time, and annually updating temperature buffering based on dynamic changes in forest structure and composition. This study is meant to contrast outcomes if realistic microclimate temperature offsets are used as the proximal drivers of forest understory processes, not to provide an actual projection of change for this forest landscape. Our empirical model is calibrated for topographically complex, temperate forest landscapes in Europe, and users in other regions should test and refine models as needed and evaluate whether tree species regeneration parameters need to be updated. Some influential drivers (e.g., moderating effects of local water balance on temperature buffering; von Arx et al. 2013; Davis et al. 2019) were less relevant in this landscape but could be considered in future model development.

### Simulating future forests

Our findings suggest that forest models should explicitly consider microclimate temperature to improve inferences about the future (De Frenne et al. 2021). Disregarding temperature buffering may lead to overestimation of extinction risks due to climate change (Lenoir et al. 2017) and underprediction of lagged biodiversity change in subcanopy forest communities (Zellweger et al. 2020). Forests may maintain favorable temperature conditions for many forest-dependent species under increasingly extreme climate change, potentially giving species more time to move to new habitats (i.e., as stepping-stones) or sustaining habitats for relatively immobile plant and animal species (i.e., as holdouts or microrefugia; Hannah et al. 2014). Because forest management alters canopy density and structure, accounting for resulting impacts on microclimate temperature can improve our understanding of how management affects forest processes from local to landscape scales (Chen et al. 1999; Menge et al. 2023). Forests cool microclimates more when macroclimate temperatures are hotter, suggesting that microclimate effects will be even more pronounced under future climate change if forest cover is maintained (De Lombaerde et al. 2022). Here, we present a new microclimate module for a freely available, process-based forest landscape model that allows us to explore a wide variety of climate, disturbance, and forest management scenarios and quantify the implications of temperature buffering on future forests and the services they provide.

## Supplementary Information

Below is the link to the electronic supplementary material.

Supplementary file1 (PDF 1565 KB)

## Data Availability

Data and code that support the findings of this study, including source code for the iLand version used in this study, are openly available at the Environmental Data Initiative: 10.6073/pasta/06059a68275f7d0c56c9055df3288aac. The individual-based forest landscape and disturbance model iLand is freely available, open source, and fully documented (https://iland-model.org/).
