# Supplementary material for: Microclimate temperature effects propagate across scales in forest ecosystems
Source: Landsc Ecol. 2025 Feb 3;40(2):37. doi: 10.1007/s10980-025-02054-8 (PMC11790809; doi:10.1007/s10980-025-02054-8)
Supplement: Supplementary file 1 — Supplementary file1 (PDF 1565 KB) [file 10980_2025_2054_MOESM1_ESM.pdf]

Microclimate temperature effects propagate across scales in forest ecosystems

Kristin H. Braziunas, Werner Rammer, Pieter De Frenne, Joan Díaz-Calafat, Per-Ola Hedwall, Cornelius Senf, Dominik Thom, Florian Zellweger, and Rupert Seidl

Supplementary materials and methods

Empirical temperature offset models

Within each forested study site, daily minimum and maximum microclimate temperature were recorded at plot centers either with Lascar Easy Log EL-USB-1 at 1 m height (Zellweger et al. 2019; Meeussen et al. 2021) or HOBO Pendant MX Water Temperature loggers at 1.2 m height (Díaz-Calafat et al. 2023). Prior to model fitting, temperature data were reviewed and cleaned to identify and remove snow days, erroneous time periods, and extreme outliers. This removed 5% of daily data from further analysis.

Snow days. We identified days when temperature loggers were covered with snow as days where the daily temperature range was < 1°C, the average maximum daily temperature was < 1°C in a 9-day moving window, and the average daily temperature range was < 2°C within the same 9-day moving window (Aalto et al. 2022; Tyystjärvi et al. 2023). Since loggers were at 1-1.2 m height, this only occurred in some winters in plots in Scandinavia. Subsequently, all days within a 5-day moving window were classified as snow days if at least one day was snow-covered (Tyystjärvi et al. 2023).

Erroneous time periods. Microclimate loggers were grouped by region to identify erroneous time periods. Anomalies were automatically detected by taking the 30-day running mean of maximum and minimum temperature for all loggers in a region and then identifying measurements that were more than three standard deviations from the mean value (*sensu* Aalto et al. 2022). We then visually evaluated all loggers with anomalies by comparing time series with other loggers in the same region and, when applicable, at the same site within a region (*sensu* Meeussen et al. 2021). If loggers exhibited sustained time periods with

anomalous values, did not align with other loggers at the same region or site, or did not exhibit expected seasonal trends, these time periods were classified as erroneous (e.g., potentially a period when the logger was uprooted or damaged) and removed from analyses.

Extreme outliers. Individual outlier values were removed based on similar criteria. Extreme outliers were identified as any values that was greater than three standard deviations from the 30-day running mean for each logger and as values that were < -50°C or > 50°C (Tyystjärvi et al. 2023). These outliers were also removed from analyses.

Predictor variables. Predictor variable selection was based on important predictors identified in previous studies and *a priori* expectations based on ecological relationships (Table 2 in main manuscript). Other predictors were considered but excluded due to high collinearity (e.g., phenology with macroclimate temperature) or duplicative information (e.g., elevation with macroclimate temperature, cold air drainage with topographic position index), unbalanced representation among the different studies (e.g., proportion deciduous or coniferous), lack of variability within the study landscape (e.g., distance to coast does not vary meaningfully within Berchtesgaden), and inadequate coverage of values in the study landscape (e.g., relatively shallower stopes in microclimate dataset and steeper slopes in Berchtesgaden). Final predictors were not strongly correlated (all bivariate Pearson's $r < 0.5$) and summary statistics by study area are included in Table S1.

Table S1. Summary data on microclimate and macroclimate temperature data collection dates and mean, standard deviation, and range of dependent and predictor variables used in empirical temperature offset models for each of the three studies and for all studies combined.

Variable	Units	**Díaz-Calafat et al. 2023** mean (sd) min-max	**Meeussen et al. 2021** mean (sd) min-max	**Zellweger et al. 2019** mean (sd) min-max	**All studies** mean (sd) min-max
Data collection					
Date range	–	Jan 2020-July 2021	June 2018-May 2020	Mar 2017-Jan 2018	2017-2021
Number of observations (monthly averages of daily values)	–	2381	4370	1004	7755
Dependent variables					
Average daily minimum microclimate temperature offset ($Tmin_{offset}$)	°C	-0.03 (1.3) -2.5-3.4	1.28 (1.59) -3.9-8.26	0.89 (1.1) -1.97-4.11	0.83 (1.56) -3.9-8.26
Average daily maximum microclimate temperature offset ($Tmax_{offset}$)	°C	0.54 (1.49) -2.88-8.78	-5.02 (4.23) -18.25-14.23	-0.83 (1.4) -4.34-3.98	-2.77 (4.21) -18.25-14.23
Predictor variables					
Average daily minimum macroclimate temperature ($Tmin_{macroclimate}$)	°C	3.26 (6.23) -12.44-15.42	5.09 (5.34) -7.5-16.5	6.28 (4.69) -5.58-13.88	4.68 (5.64) -12.44-16.5
Average daily maximum macroclimate temperature ($Tmax_{macroclimate}$)	°C	11.48 (7.55) -5.37-24.96	22.54 (10.66) -0.52-44.93	14.83 (6.83) 0.89-28.07	18.15 (10.65) -5.37-44.93
Northness	dim[-1,1]	-0.22 (0.69) -1-1	-0.47 (0.67) -1-1	-0.11 (0.73) -1-1	-0.35 (0.7) -1-1
Topographic position index (TPI)	m	-0.02 (4.45) -8.71-12.33	-3.33 (24.71) -105.43-63.2	4.94 (13.68) -15.98-66.8	-1.24 (19.55) -105.43-66.8
Leaf area index (LAI)	$m^2 m^{-2}$	2.65 (1.33) 0.37-6.33	2.53 (1.35) 0.3-7.74	3.39 (1.54) 0.52-9.44	2.67 (1.4) 0.3-9.44
Shade tolerance (STol)	dim[1,5]	2.53 (0.71) 1-3.5	3.07 (0.88) 1.06-5	3.68 (0.68) 2-5	2.98 (0.88) 1-5

Daily minimum and maximum macroclimate temperature for each site was recorded from either nearby weather stations (Zellweger et al. 2019; Díaz-Calafat et al. 2023) or from an identical temperature logger installed nearby in open conditions (Meeussen et al. 2021). Macroclimate loggers installed in open conditions were also quality checked, and snow days and extreme outliers were identified and excluded as described above. Temperature offset was

then calculated as microclimate minus macroclimate temperature for daily minimum and maximum values (Equations S1-S2).

$$Tmin_{offset} = Tmin_{microclimate} - Tmin_{macroclimate} \quad \text{(Eq. S1)}$$

$$Tmax_{offset} = Tmax_{microclimate} - Tmax_{macroclimate} \quad \text{(Eq. S2)}$$

Negative offset values indicate that microclimate temperatures are cooler underneath the forest canopy relative to macroclimate temperatures, whereas positive values indicate subcanopy temperatures are warmer. Daily temperature offsets were averaged for each month, and only months with at least 15 daily observations were included in model fitting.

Topographic predictors were derived from field plot coordinates and a 25 m resolution digital elevation model (EU-DEM 2016). Forest structure and composition predictors were calculated from forest inventory data including individual tree species and diameter at breast height (DBH) for all trees with DBH > 7.5 cm in a 9 m radius plot centered on the location of the microclimate logger. We quantified plot-level variables using previously compiled and tested species-specific trait values for foliage biomass allometry, specific leaf area, and shade tolerance for Central European tree species simulated in iLand (Seidl et al. 2012; Thom et al. 2017, 2022). Species not present in this dataset were assigned biomass allometrics from a morphologically similar species (based on Falster et al. 2015; Forrester et al. 2017). Additional data on shade tolerance was procured from Niinemets & Valladares (2006) via the TRY Plant Trait Database (Kattge et al. 2020).

A random intercept effect for study (n = 3) was included to account for variance due to methodological or other differences among studies (e.g., different microclimate temperature sensors, macroclimate data sources, measurement height, and data cleaning processes) not explained by fixed effects. This assumed that study was independent of the fixed effects. We evaluated this assumption by testing for multicollinearity among all predictors using generalized variance inflation factors (GVIF), which when rescaled based on degrees of freedom are suitable for evaluating correlation strength for categorical predictors with more

than two levels (Fox and Monette 1992; Fox 2016). The squared scaled GVIF is identical to the variance inflation factor for continuous variables and interpreted using the same ranges of values to assess correlation strength. We further evaluated the inclusion of study by comparing residual boxplots between models with or without study as a predictor; the inclusion of study as a random effect removed directional trends in median residual values, although some unequal variance remained (Figure S1).

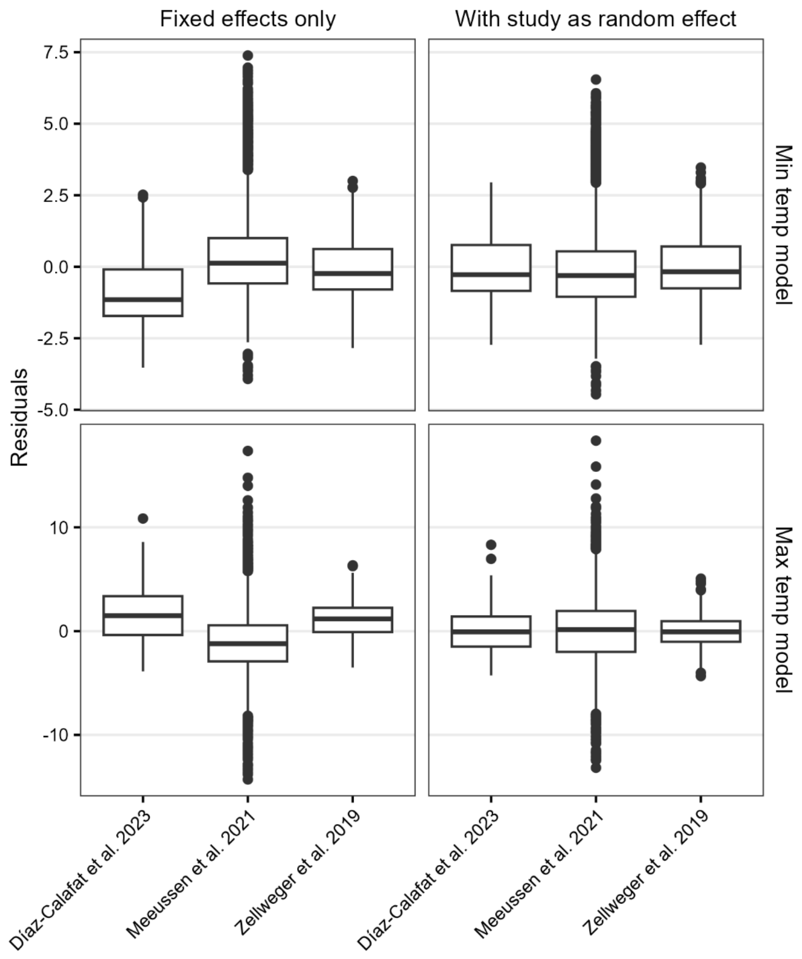

Figure S1. Boxplots showing trends and variability in residual values among different studies for linear models with only fixed effects (study not included as a predictor; left column) and for linear mixed effects models when study was included as a random intercept effect (right column). Top row: minimum temperature offset model, Bottom row: maximum temperature offset model.

Model diagnostics and decision-making. Linear mixed effects model diagnostics based on residual and quantile-quantile plots showed slight deviations from assumptions of

normality, linearity, and equal variance (Figures S2-S3). We used and considered multiple approaches for improving model assumptions, including removing erroneous values and outliers (described above), using the monthly average of daily minimum and maximum temperatures rather than the daily values, adding more predictors, transforming predictors, including or excluding study as a random effect, and fitting separate models for each study. Adding new or transforming predictors did not improve assumptions, but using the monthly average of daily values and including study as a random effect did improve assumptions (e.g., see Figure S1 above). Separate models fit to each study showed that assumption violations varied by dependent variable and by study (Figures S4-S5).

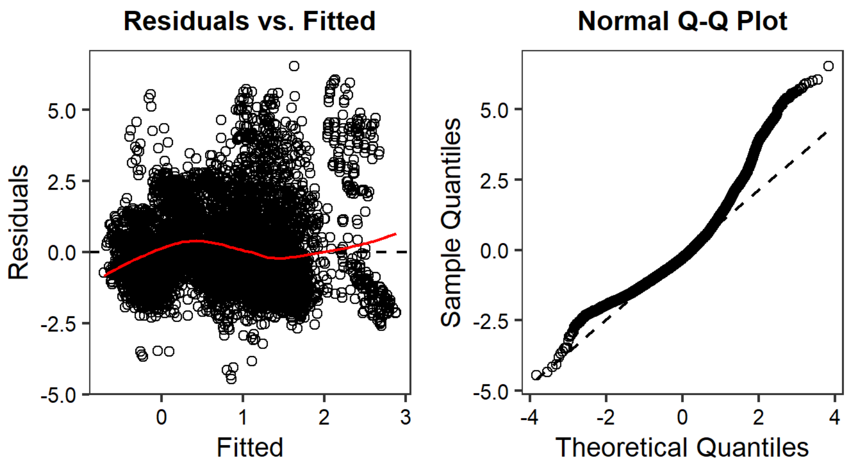

Figure S2. Final linear mixed effects model diagnostics, including residuals versus fitted values and quantile-quantile plot, for minimum temperature offset.

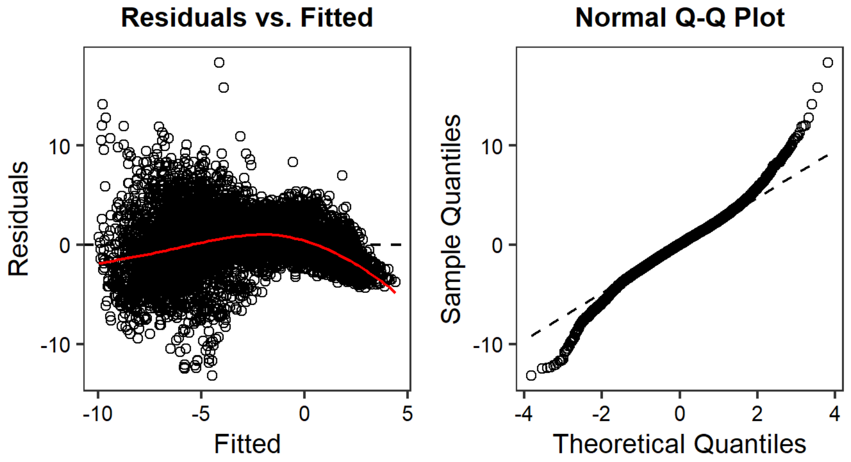

Figure S3. Final linear mixed effects model diagnostics, including residuals versus fitted values and quantile-quantile plot, for maximum temperature offset.

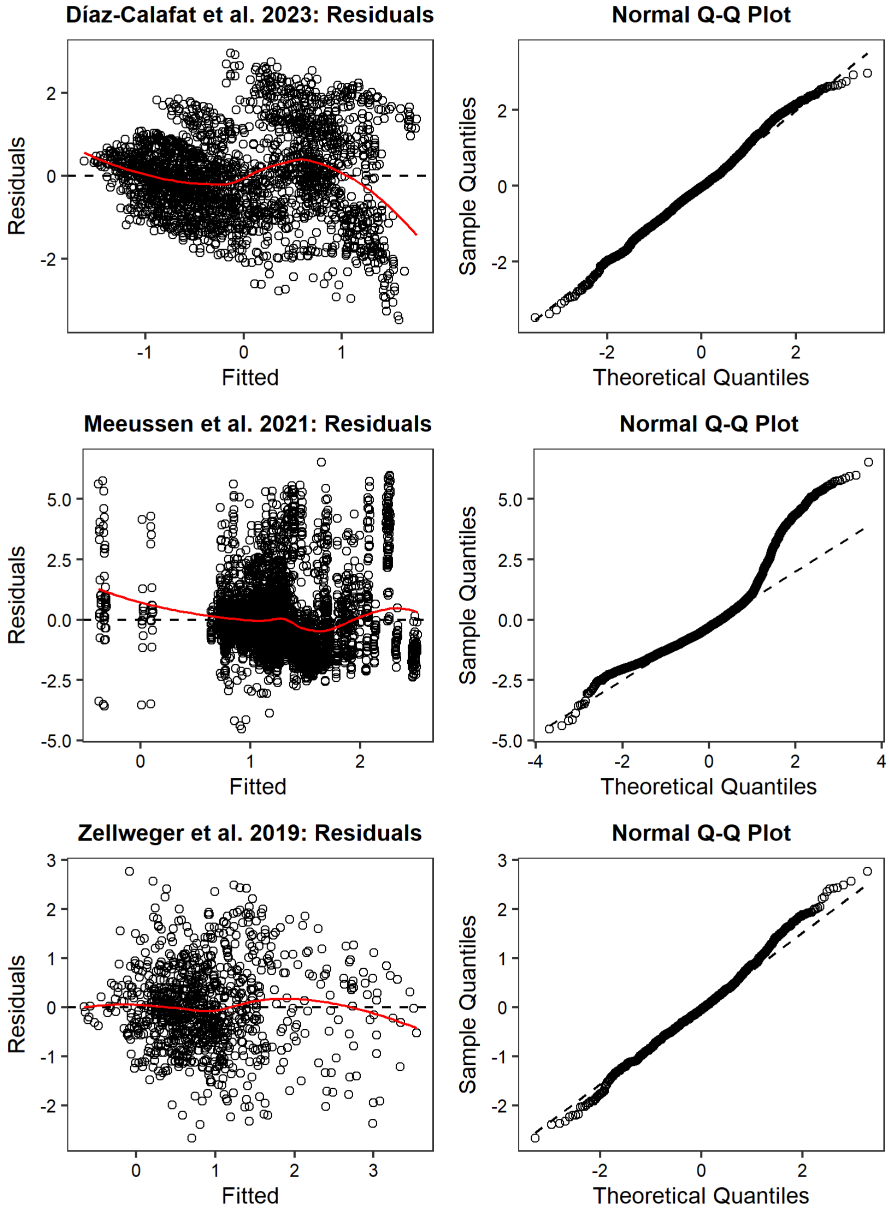

Figure S4. Linear model diagnostics, including residuals versus fitted values (left column) and quantile-quantile plots (right column), for minimum temperature offset models fit to each study separately (rows).

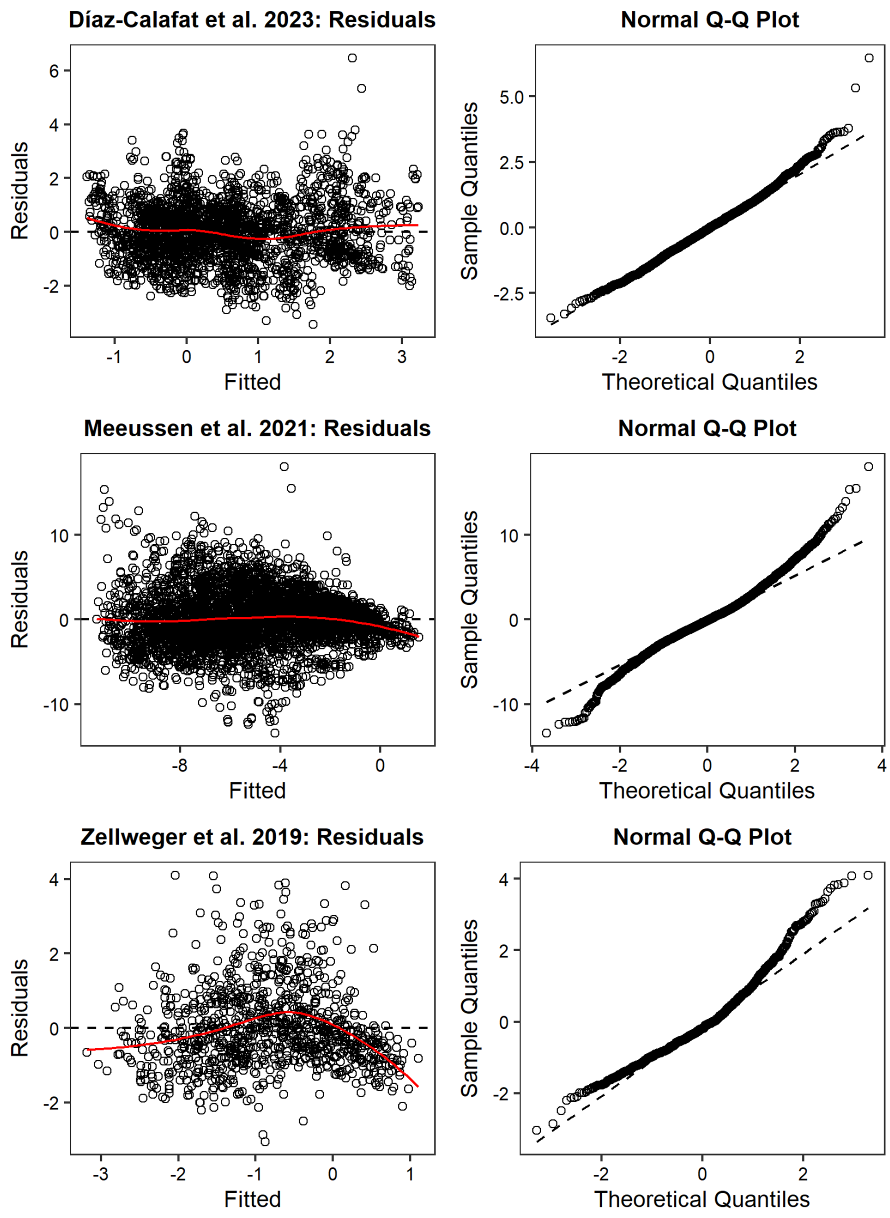

Figure S5. Linear model diagnostics, including residuals versus fitted values (left column) and quantile-quantile plots (right column), for maximum temperature offset models fit to each study separately (rows).

We considered model goals and the balance of generality, realism, and precision in final model decision-making (Levins 1966). For the purposes of implementing microclimate temperature offsets in a process-based forest landscape simulation model, we prioritized simplicity, generality, and ecological realism at the expense of additional model precision in the fit of the statistical model. Thus, we chose a linear model for simplicity and included all data despite assumption violations to improve generality. We further considered ecological

realism in model evaluations, such as expectations from the literature and biophysical principles for relationships between fixed effects and predicted offsets, seasonal and forest type variation in predicted offsets, and comparisons with independent data. Finally, we considered and took steps to constrain the potential range of predicted values. Predictions tended to be more conservative relative to observations (i.e., overpredicted at low extremes and underpredicted at high extremes; Figure S6a-b). We truncated all predictor values to the maximum and minimum values used in model fitting to avoid extrapolating beyond the range of values used to train the models.

Final models. Final linear mixed effects models predicting the monthly average of daily minimum ($R^2_c = 0.24$, $R^2_m = 0.07$) and maximum ($R^2_c = 0.47$, $R^2_m = 0.29$) temperature offsets were fit to n = 7,755 observations in n = 497 plots (Equations S3-S4; Tables 3-4; Figure S6). Note that Equations show fixed effects and the average intercept across studies; the random effect (study) would be reflected by having a different intercept for each study.

$$Tmin_{offset} = 1.4570 - 0.0248 \times Tmin_{macroclimate} + 0.2627 \times Northness + 0.0158 \times TPI + 0.0227 \times LAI - 0.2031 \times STol \quad \text{(Eq. S3)}$$

$$Tmax_{offset} = 0.9767 - 0.1932 \times Tmax_{macroclimate} - 0.5729 \times Northness + 0.0140 \times TPI - 0.3948 \times LAI + 0.4419 \times STol \quad \text{(Eq. S4)}$$

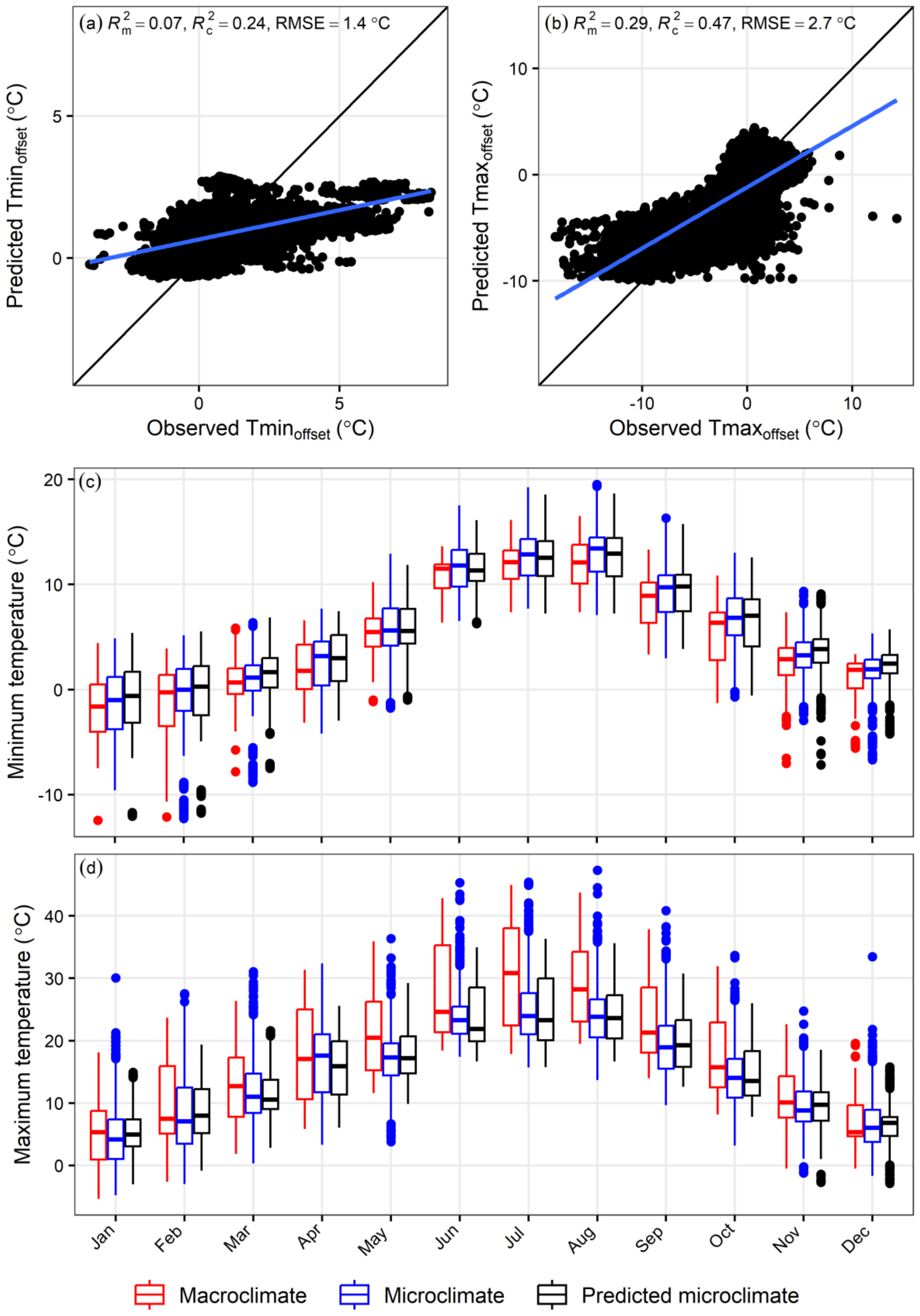

Figure S6. (a-b) Predicted versus observed average daily minimum (a) and maximum (b) microclimate temperature offset from linear mixed effects models fit to monthly averages (*n* = 7,755 observations from three studies). Black line is 1:1 line, blue line is linear fit. (c-d) Temporal variability in macroclimate, microclimate, and predicted microclimate for average daily minimum (c) and maximum (d) temperature.

Initial conditions for iLand simulations

Initial forest structure and species composition was mapped from 3,559 regularly spaced forest inventory plots and a forest type map. Daily climate (1980-2009) was derived from bias corrected dynamic regional climate projections (Warscher et al. 2019) using 35 local weather stations and statistically downscaled to 100 m resolution accounting for the effect of elevation. Historical wind event speed, direction, and day of year were modeled from regional meteorological station measurements, and simulated wind and bark beetle disturbances aligned well with past observations (Thom et al. 2022). Soil texture and fertility were mapped from regional data (Konnert 2004), and topographic variables were derived from a digital elevation model (EU-DEM 2016) downscaled from 25 to 10 m resolution using bilinear interpolation.

Analyses across scales

At local scales, we compared microclimate temperature effects on forest processes in dense forested stands, defined as having overstory LAI > 4 m^2 m^{-2} (von Arx et al. 2013). Using the first 30 years per simulation replicate, we computed the annual mean value for indicators of each of the three focal processes: heterotrophic respiration (Mg C ha^{-1}) as an indicator of decomposition, number of completed beetle generations as an indicator of bark beetle development rates, and tree regeneration density (total and species-specific stems ha^{-1} for stems < 4 m height) as an indicator of tree establishment.

At mesoscales, we evaluated disturbance effects on the same decomposition, bark beetle, and establishment indicators as the post- minus pre-disturbance mean value for each disturbance patch in microclimate and macroclimate simulations. Stands were considered disturbed if at least half of the 1-ha area experienced a bark beetle or windthrow event over the first 10 simulation years, to account for multi-year bark beetle spread or wind-beetle interactions. Patches (minimum size = 1 ha) were then classified using the 8-neighbor rule. Pre-disturbance indicators were calculated for simulation year 0 and post-disturbance for year

15 (i.e., 5-15 years since disturbance) based on forest recovery rates and the timing of peak microclimate temperature buffering in this landscape (Vandewiele et al. 2023).

Mesoscale differences in tree regeneration were also evaluated for six representative species that varied in elevational range and temperature sensitivity. These included beech and silver fir [submontane-montane zone, warm-preferring with Ellenberg Indicator Value (EIV) for temperature = 5; Ellenberg & Leuschner, 2010], spruce and Swiss stone pine (subalpine, cold-preferring with EIV = 3 and 2, respectively), and sycamore maple (*Acer pseudoplatanus* L.) and larch (montane and subalpine, respectively, temperature indifferent). The elevational regeneration range for each species was represented with 100 m bands centered on the approximate lower bound, median, and upper bound of its elevational regeneration distribution in the Bavarian Alps (Ewald 2012). Lower bounds were excluded from analysis if they fell below the minimum elevation in the Berchtesgaden landscape (~600 m). Variable effects of microclimate along the elevational regeneration range were quantified as the relative difference in stem density between microclimate and macroclimate simulations for each species and elevation band, averaged across the first 30 simulation years.

At the landscape scale, we compared cumulative net ecosystem productivity (NEP), total carbon, carbon pools, cumulative disturbance mortality, and tree species composition (trees > 4 m height) based on basal area after 1,000 years of forest development with or without microclimate temperature buffering. Indicators that were not cumulative (carbon pools and species basal area) were averaged over the last 30 simulation years. We also compared relative differences in landscape scale indicators between the first and last 30 simulation years (here, annual rather than cumulative values were used for NEP and disturbance) and with the local scale indicators described above to evaluate how microclimate effects changed over time and across scales.

Sensitivity analysis

A sensitivity analysis was performed to determine which process most strongly contributed to landscape scale change in cumulative NEP, total carbon, and individual carbon pools when driven by microclimate rather than macroclimate temperature. To determine relative effects, microclimate temperature buffering was turned "on" or "off" for each of the three processes (decomposition, bark beetle development, tree establishment), and simulations were run for all combinations (n = 10 replicates of each 2^3 processes = 80 total replicates). For each replicate, forest development was simulated for 30 years under historical climate, random sequences of wind events, and dynamic bark beetle disturbances starting from contemporary forest conditions in Berchtesgaden National Park. Cumulative NEP at year 30 and average carbon pools were normalized by subtracting the corresponding macroclimate replicate (microclimate = "off" for all processes) and dividing by the range of simulation means (i.e., the range of all eight process combinations after averaging across the 10 replicates, so that mean differences will be within +/-1).

Cumulative NEP, total carbon, and all carbon pools except live C were most sensitive to microclimate temperature buffering effects on decomposition (Figure S7). Dampened bark beetle development rates due to microclimate buffering resulted in the greatest increases in live C and decreases in dead woody C that partially offset gains from reduced decomposition.

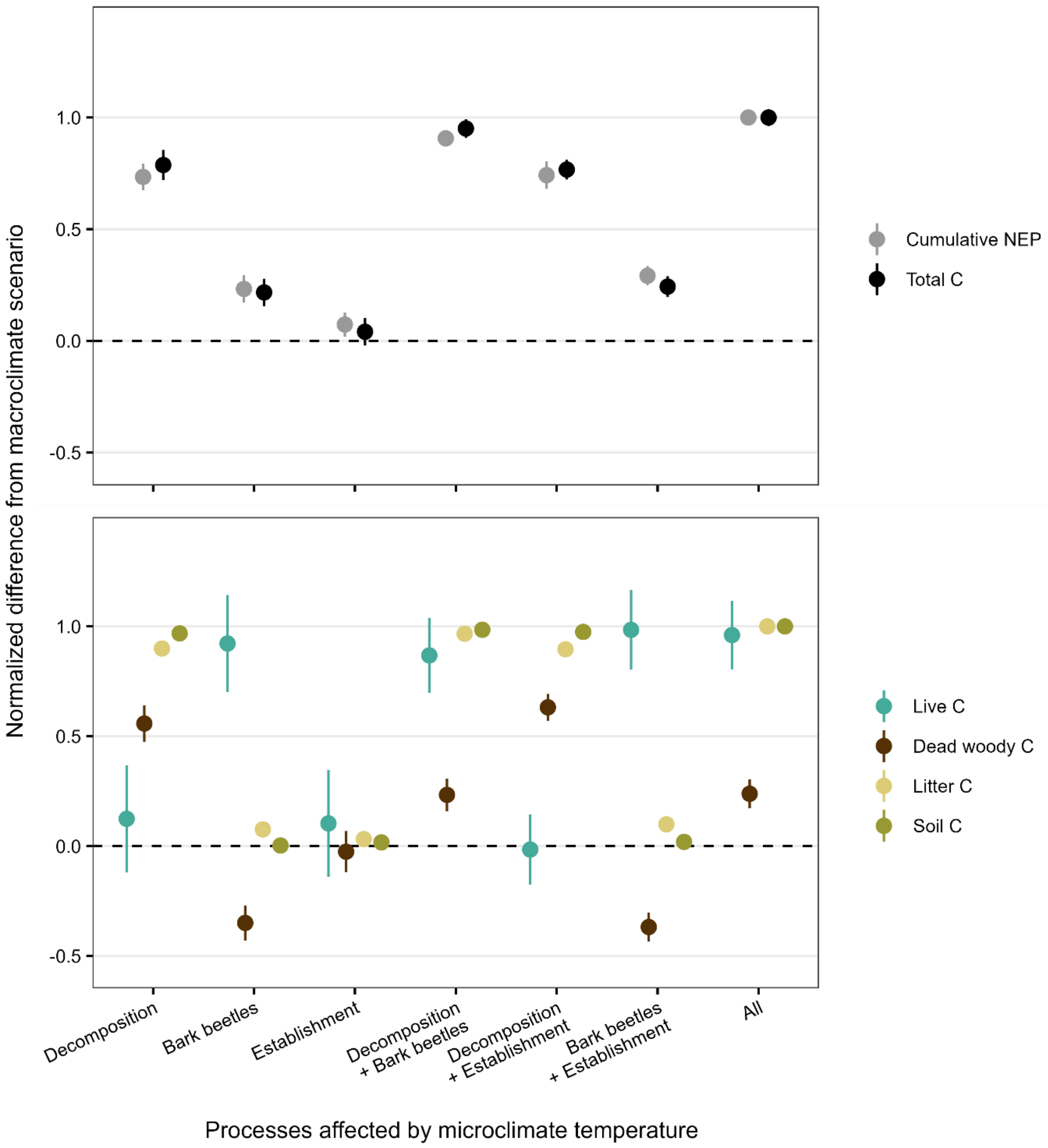

Figure S7. Sensitivity analysis showing the effect of driving simulations with microclimate instead of macroclimate for all combinations of three forest processes on (a) cumulative net ecosystem productivity (NEP) and total carbon (C) or (b) different carbon pools. X axis shows which processes are driven by microclimate. Points and ranges are derived from $n = 10$ replicates of each and show the mean change (point) and two standard errors (range). All values have been normalized by subtracting the corresponding macroclimate replicate value and dividing by the range of simulation means [i.e., the range of all eight process combinations after averaging across the 10 replicates, so that mean differences (points) will be within +/-1].

Software

All statistical analyses were performed and, with the exception of Figure 1, all figures were created using R (R Core Team 2024) version 4.3.2. We specifically used the packages car (Fox and Weisberg 2019), corrplot (Wei and Simko 2021), cowplot (Wilke 2020), ggnewscale (Campitelli 2023), ggpubr (Kassambara 2023), landscapemetrics (Hesselbarth et al. 2019), lme4 (Bates et al. 2015), lmerTest (Kuznetsova et al. 2017), lubridate (Grolemund and Wickham 2011), ModelMetrics (Hunt 2020), MuMIn (Bartoń 2023), openxlsx (Schauberger and Walker 2023), plotrix (Lemon 2006), RSQLite (Müller et al. 2023), sf (Pebesma 2018; Pebesma and Bivand 2023), terra (Hijmans 2023), tidyverse (Wickham et al. 2019), and zoo (Zeileis and Grothendieck 2005). Figure color schemes were derived from Color Brewer 2.0 (Brewer and Harrower 2013), khroma (Frerebeau 2023), and Paul Tol's Color Schemes (Tol 2023).

References

Aalto J, Tyystjärvi V, Niittynen P, et al (2022) Microclimate temperature variations from boreal forests to the tundra. Agricultural and Forest Meteorology 323:109037. https://doi.org/10.1016/j.agrformet.2022.109037

Bartoń K (2023) MuMIn: Multi-model inference. R package version 1.47.5. https://CRAN.R-project.org/package=MuMIn

Bates D, Mächler M, Bolker BM, Walker SC (2015) Fitting linear mixed-effects models using lme4. Journal of Statistical Software 67:1–48. https://doi.org/10.18637/jss.v067.i01

Brewer C, Harrower M (2013) Color Brewer 2.0: Color advice for cartography. https://colorbrewer2.org/. Accessed 31 Jan 2024

Campitelli E (2023) ggnewscale: Multiple fill and colour scales in ggplot2. R package version 0.4.9. https://CRAN.R-project.org/package=ggnewscale

Díaz-Calafat J, Uria-Diez J, Brunet J, et al (2023) From broadleaves to conifers: The effect of tree composition and density on understory microclimate across latitudes. Agricultural and Forest Meteorology 341:109684. https://doi.org/10.1016/j.agrformet.2023.109684

Ellenberg H, Leuschner C (2010) Vegetation Mitteleuropas mit den Alpen. In ökologischer, dynamischer und historischer Sicht, 6th edn. UTB, Stuttgart

EU-DEM (2016) European Digital Elevation Model (EU-DEM), version 1.1. http://land.copernicus.eu/pan-european/satellite-derived-products/eu-dem/eu-dem-v1.1/view. Accessed 31 Jul 2023

Ewald J (2012) Vegetation databases provide a close-up on altitudinal tree species distribution in the Bavarian Alps. Vegetation databases for the 21st century–Biodiversity & Ecology 4:41–48

Falster DS, Duursma RA, Ishihara MI, et al (2015) BAAD: a Biomass And Allometry Database for woody plants. Ecology 96:1445–1445. https://doi.org/10.1890/14-1889.1

Forrester DI, Tachauer IHH, Annighoefer P, et al (2017) Generalized biomass and leaf area allometric equations for European tree species incorporating stand structure, tree age and climate. Forest Ecology and Management 396:160–175. https://doi.org/10.1016/j.foreco.2017.04.011

Fox J (2016) Applied regression analysis and generalized linear models, Third edition. SAGE, Los Angeles

Fox J, Monette G (1992) Generalized collinearity diagnostics. Journal of the American Statistical Association 87:178–183. https://doi.org/10.1080/01621459.1992.10475190

Fox J, Weisberg S (2019) An R companion to applied regression. Sage, Thousand Oaks, CA

Frerebeau N (2023) khroma: Colour schemes for scientific data visualization. R package version 1.11.0. https://packages.tesselle.org/khroma

Grolemund G, Wickham H (2011) Dates and times made easy with lubridate. Journal of Statistical Software 40:1–25. https://doi.org/10.18637/jss.v040.i03

Hesselbarth MHK, Sciaini M, With KA, et al (2019) landscapemetrics: An open-source R tool to calculate landscape metrics. Ecography 42:1648–1657. https://doi.org/10.1111/ecog.04617

Hijmans RJ (2023) terra: Spatial data analysis. R package version 1.7-55. https://CRAN.R-project.org/package=terra

Hunt T (2020) ModelMetrics: Rapid calculation of model metrics. R package version 1.2.2.2. https://CRAN.R-project.org/package=ModelMetrics

Kassambara A (2023) ggpubr: ggplot2 based publication ready plots. R package version 0.6.0. https://CRAN.R-project.org/package=ggpubr

Kattge J, Bönisch G, Díaz S, et al (2020) TRY plant trait database – enhanced coverage and open access. Global Change Biology 26:119–188. https://doi.org/10.1111/gcb.14904

Konnert V (2004) Standortkarte Nationalpark Berchtesgaden. Nationalpark Berchtesgaden, Forschungsbericht 49, Berchtesgaden, DE

Kuznetsova A, Brockhoff PB, Christensen RHB (2017) lmerTest Package: Tests in Linear Mixed Effects Models. Journal of Statistical Software 82:1–26. https://doi.org/10.18637/jss.v082.i13

Lemon J (2006) Plotrix: A package in the red light district of R. R-News 6:8–12

Levins R (1966) The strategy of model building in population biology. American Scientist 54:421–431

Meeussen C, Govaert S, Vanneste T, et al (2021) Microclimatic edge-to-interior gradients of European deciduous forests. Agricultural and Forest Meteorology 311:108699. https://doi.org/10.1016/j.agrformet.2021.108699

Müller K, Wickham H, James DA, Falcon S (2023) RSQLite: "SQLite" interface for R. R package version 2.3.3. https://CRAN.R-project.org/package=RSQLite

Niinemets Ü, Valladares F (2006) Tolerance to shade, drought, and waterlogging of temperate northern hemisphere trees and shrubs. Ecological Monographs 76:521–547. https://doi.org/10.1890/0012-9615(2006)076[0521:TTSDAW]2.0.CO;2

Pebesma E (2018) Simple features for R: Standardized support for spatial vector data. R Journal 10:439–446. https://doi.org/10.32614/rj-2018-009

Pebesma E, Bivand R (2023) Spatial Data Science: With Applications in R., 1st Edition. Chapman and Hall/CRC, Boca Raton, FL

R Core Team (2024) R: A language and environment for statistical computing. Vienna, Austria

Schauberger P, Walker A (2023) openxlsx: Read, write and edit xlsx files. R package version 4.2.5.2. https://CRAN.R-project.org/package=openxlsx

Seidl R, Rammer W, Scheller RM, Spies TA (2012) An individual-based process model to simulate landscape-scale forest ecosystem dynamics. Ecological Modelling 231:87–100. https://doi.org/10.1016/j.ecolmodel.2012.02.015

Thom D, Rammer W, Dirnböck T, et al (2017) The impacts of climate change and disturbance on spatio-temporal trajectories of biodiversity in a temperate forest landscape. Journal of Applied Ecology 54:28–38. https://doi.org/10.1111/1365-2664.12644

Thom D, Rammer W, Laux P, et al (2022) Will forest dynamics continue to accelerate throughout the 21st century in the Northern Alps? Global Change Biology 28:3260–3274. https://doi.org/10.1111/gcb.16133

Tol P (2023) Paul Tol's Notes: Colour schemes and templates. https://personal.sron.nl/~pault/. Accessed 31 Jan 2024

Tyystjärvi VA, Niittynen P, Kemppinen J, et al (2023) Variability and drivers of winter near-surface temperatures over boreal and tundra landscapes. EGUsphere 2023:1–24. https://doi.org/10.5194/egusphere-2023-576

Vandewiele M, Geres L, Lotz A, et al (2023) Mapping spatial microclimate patterns in mountain forests from LiDAR. Agricultural and Forest Meteorology 341:109662. https://doi.org/10.1016/j.agrformet.2023.109662

von Arx G, Pannatier EG, Thimonier A, Rebetez M (2013) Microclimate in forests with varying leaf area index and soil moisture: Potential implications for seedling

establishment in a changing climate. Journal of Ecology 101:1201–1213. https://doi.org/10.1111/1365-2745.12121

Warscher M, Wagner S, Marke T, et al (2019) A 5 km resolution regional climate simulation for Central Europe: Performance in high mountain areas and seasonal, regional and elevation-dependent variations. Atmosphere 10:682. https://doi.org/10.3390/atmos10110682

Wei T, Simko V (2021) corrplot: Visualization of a correlation matrix. R package version 0.92. https://github.com/taiyun/corrplot

Wickham H, Averick M, Bryan J, et al (2019) Welcome to the Tidyverse. Journal of Open Source Software 4:1686. https://doi.org/10.21105/joss.01686

Wilke CO (2020) cowplot: Streamlined plot theme and plot annotations for ggplot2. R package version 1.1.1. https://CRAN.R-project.org/package=cowplot

Zeileis A, Grothendieck G (2005) zoo: S3 Infrastructure for Regular and Irregular Time Series. Journal of Statistical Software 14:1–27. https://doi.org/10.18637/jss.v014.i06

Zellweger F, Coomes D, Lenoir J, et al (2019) Seasonal drivers of understorey temperature buffering in temperate deciduous forests across Europe. Global Ecology and Biogeography 28:1774–1786. https://doi.org/10.1111/geb.12991

Supplementary tables and figures

Table S2. Summary data on simulated maximum, mean, and minimum temperature offsets; macroclimate and microclimate temperature; and temperature offset predictors in Berchtesgaden National Park using the newly developed microclimate module in iLand, based on contemporary forest conditions and a year with average historical climate conditions. Summaries present mean, standard deviation (sd), and range of annual values across the entire landscape (*n* = 864,466 observations at 10 m spatial resolution).

**Variable**	**Units**	**Summary statistics**<br>mean (sd)<br>min-max
Temperature offsets		
Annual average of daily minimum microclimate temperature offset ($Tmin_{offset}$)	°C	0.81 (0.82)<br>-1.61-2.59
Annual average of daily mean microclimate temperature offset	°C	0.05 (0.85)<br>-2.86-2.41
Annual average of daily maximum microclimate temperature offset ($Tmax_{offset}$)	°C	-0.70 (1.11)<br>-5.32-3.60
Macroclimate temperature		
Annual average of daily minimum macroclimate temperature ($Tmin_{macroclimate}$)	°C	3.51 (1.51)<br>-0.83-6.43
Annual average of daily mean macroclimate temperature (mean annual temperature)	°C	5.68 (1.60)<br>1.24-8.97
Annual average of daily maximum macroclimate temperature ($Tmax_{macroclimate}$)	°C	7.84 (1.69)<br>3.31-11.51
Microclimate temperature		
Annual average of daily minimum microclimate temperature ($Tmin_{microclimate}$)	°C	4.32 (1.41)<br>-1.07-8.00
Annual average of daily mean microclimate temperature (mean annual microclimate temperature)	°C	5.73 (1.32)<br>0.87-9.99
Annual average of daily maximum microclimate temperature ($Tmax_{microclimate}$)	°C	7.14 (1.41)<br>1.73-12.56
Other predictor variables		
Northness	dim[-1,1]	0.27 (0.64)<br>-1-1
Topographic position index (TPI)	m	-8.95 (47.19)<br>-105-67
Leaf area index (LAI)	$m^2 m^{-2}$	2.76 (2.09)<br>0.3-9.4
Shade tolerance (STol)	dim[1,5]	2.73 (1.02)<br>1-5

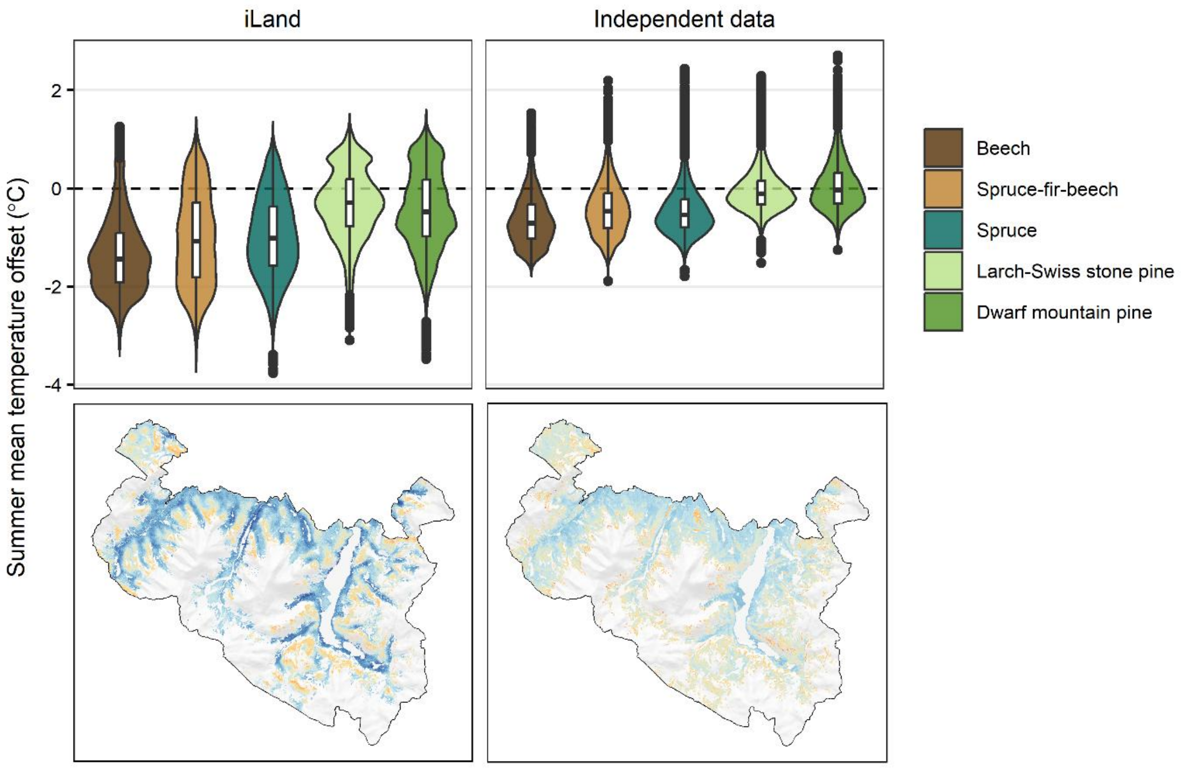

Figure S8. Comparison between simulated summer mean temperature offsets at ~1 m height in Berchtesgaden National Park using the newly developed microclimate module in iLand (left column) and temperature offset maps generated for this landscape using independent microclimate data measured at 15 cm height and mapped with LiDAR (right column; Vandewiele et al., 2023). Simulated offsets were derived at 10 m resolution (n = 864,466 observations) based on contemporary 2020 forest conditions and a year with average historical climate conditions (1988, 5.7 °C mean annual temperature). Independent data were mapped at 20 m resolution based on temperature and LiDAR data collected in 2021 (n = 229,432 observations). Corresponding values from the independent dataset were extracted using cell centroid locations from the simulated dataset, and correlation was moderately positive (Spearman's ρ = 0.47; n = 832,130 observations after removing NA values). (top row) Violin and boxplots showing summer mean minimum temperature offsets by forest type. (bottom row) Maps of summer mean temperature offsets. Mapped values are truncated to -3 and 3 to improve comparison and visualization. Temperature offsets are defined as microclimate minus macroclimate temperature.

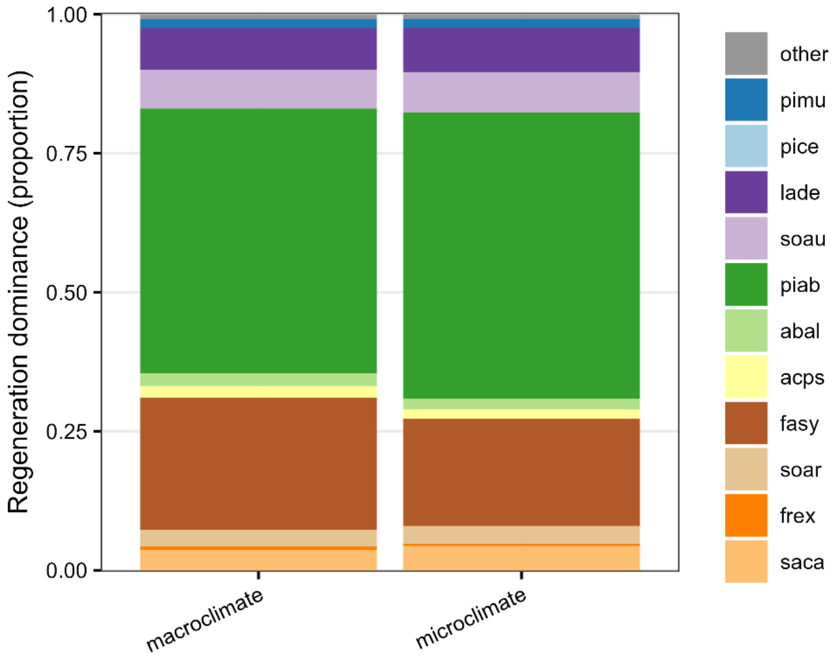

Figure S9. Tree regeneration species composition in dense forested stands (overstory LAI > 4) in microclimate versus macroclimate simulations. Stacked bars show mean proportion of total tree regeneration density for a given species across 10 simulation replicates, based on stem counts for stems < 4 m in height. Species are ordered based on whether they tend to occur at higher (pimu) to lower (saca) elevations. Species codes: pimu, *Pinus mugo*; pice, *Pinus cembra*; lade, *Larix decidua*; soau, *Sorbus aucuparia*; piab, *Picea abies*; abal, *Abies alba*; acps, *Acer pseudoplatanus*; fasy, *Fagus sylvatica*; soar, *Sorbus aria*; frex, *Fraxinus excelsior*; saca, *Salix caprea*.

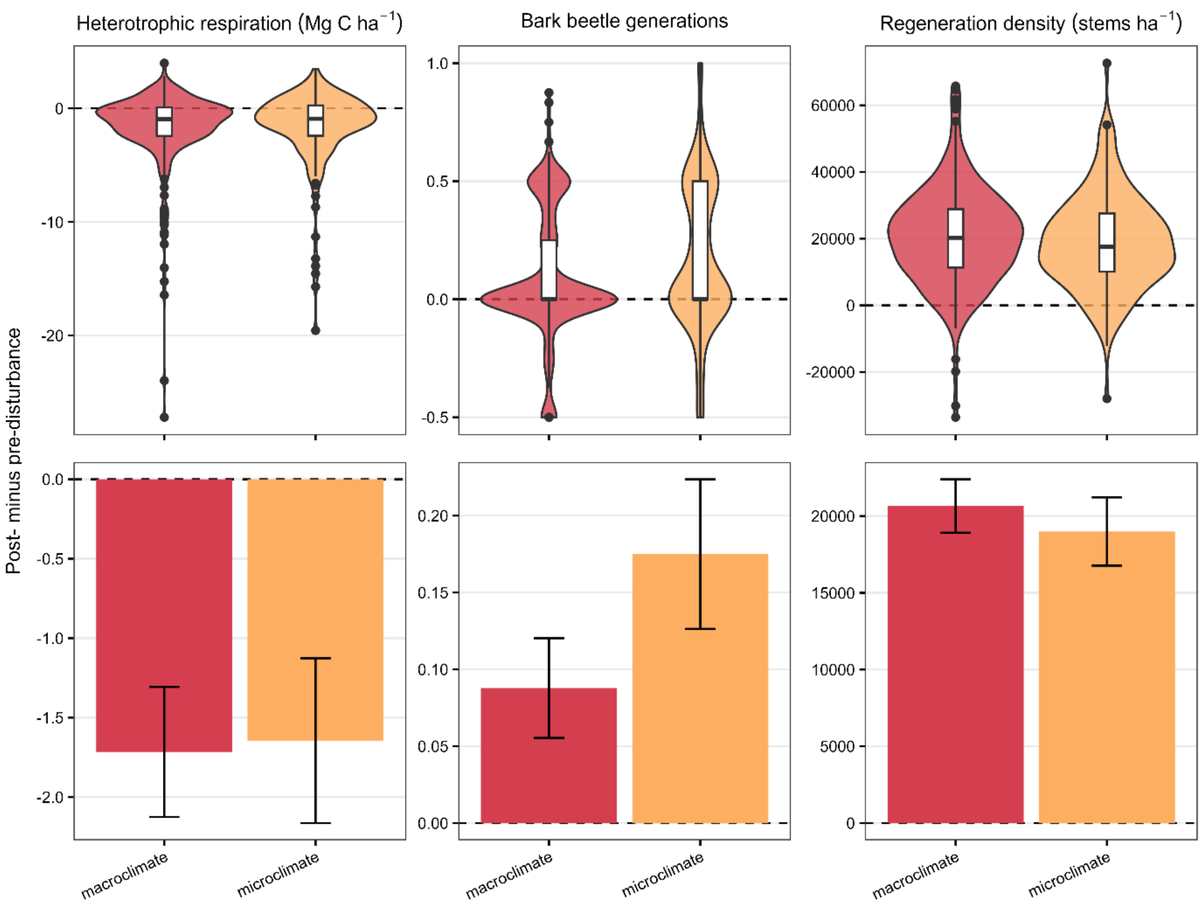

Figure S10. Disturbance effects (post- minus pre-disturbance values for each patch) on forest process indicators for microclimate and macroclimate simulations. Disturbance patches were delineated based on bark beetle and wind events occurring within the first 10 years of each simulation replicate, using the 8-neighbor rule. Pre-disturbance values were from simulation year 0, and post-disturbance values from simulation year 15 (5-15 years since disturbance). (top row) Violin and boxplots show the distribution of values across all disturbance patches (n = 283 for macroclimate, n = 165 for microclimate). (bottom row) Mean values (bars) and two standard errors (error bars) across all patches.

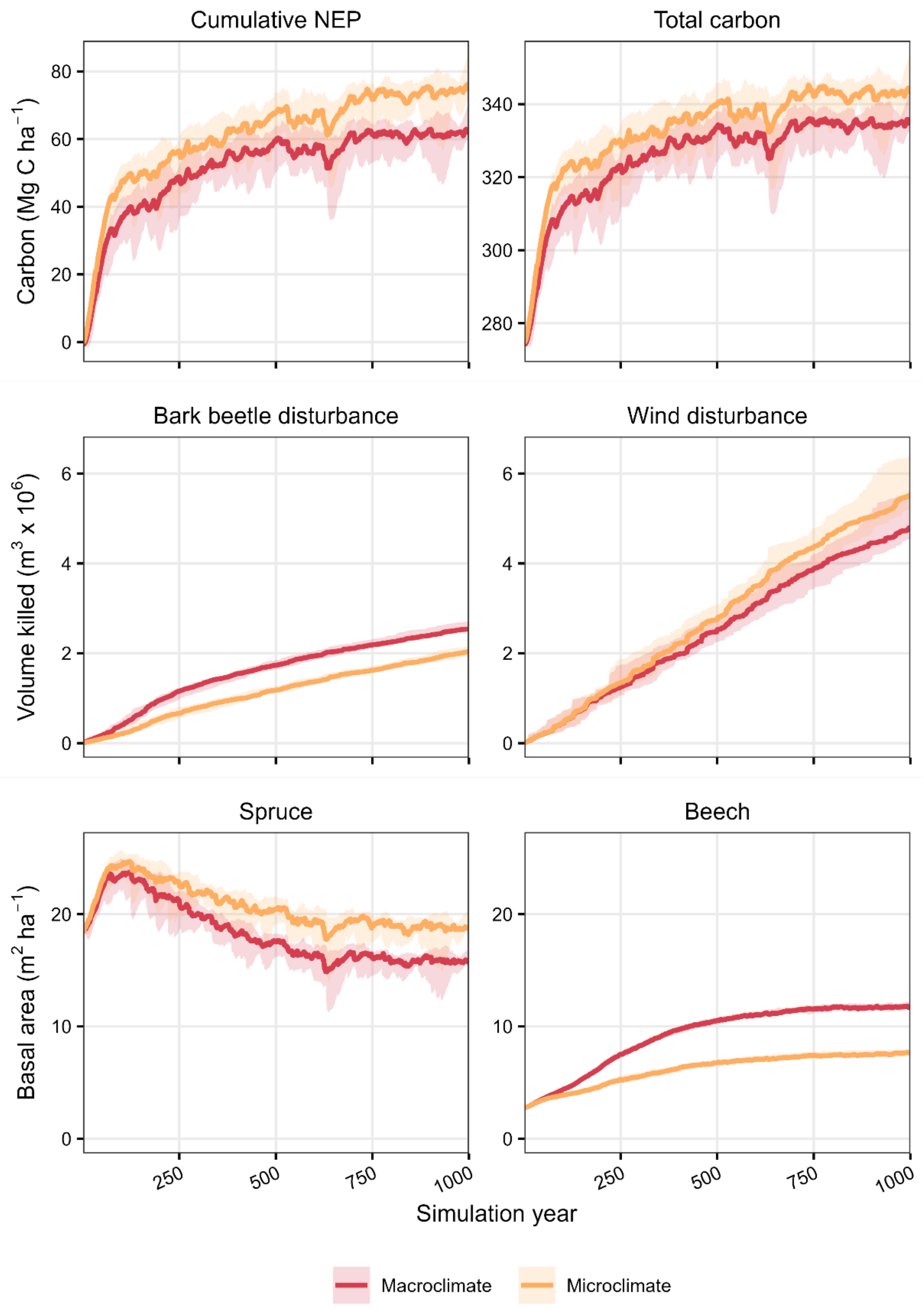

Figure S11. Landscape scale trajectories over 1,000 simulation years for cumulative net ecosystem productivity (NEP), total carbon, cumulative disturbance mortality due to bark beetles and wind, and basal area for spruce and beech, with or without microclimate temperature buffering effects. Lines are median values and shading shows 5th to 95th percentile values across 10 simulation replicates.

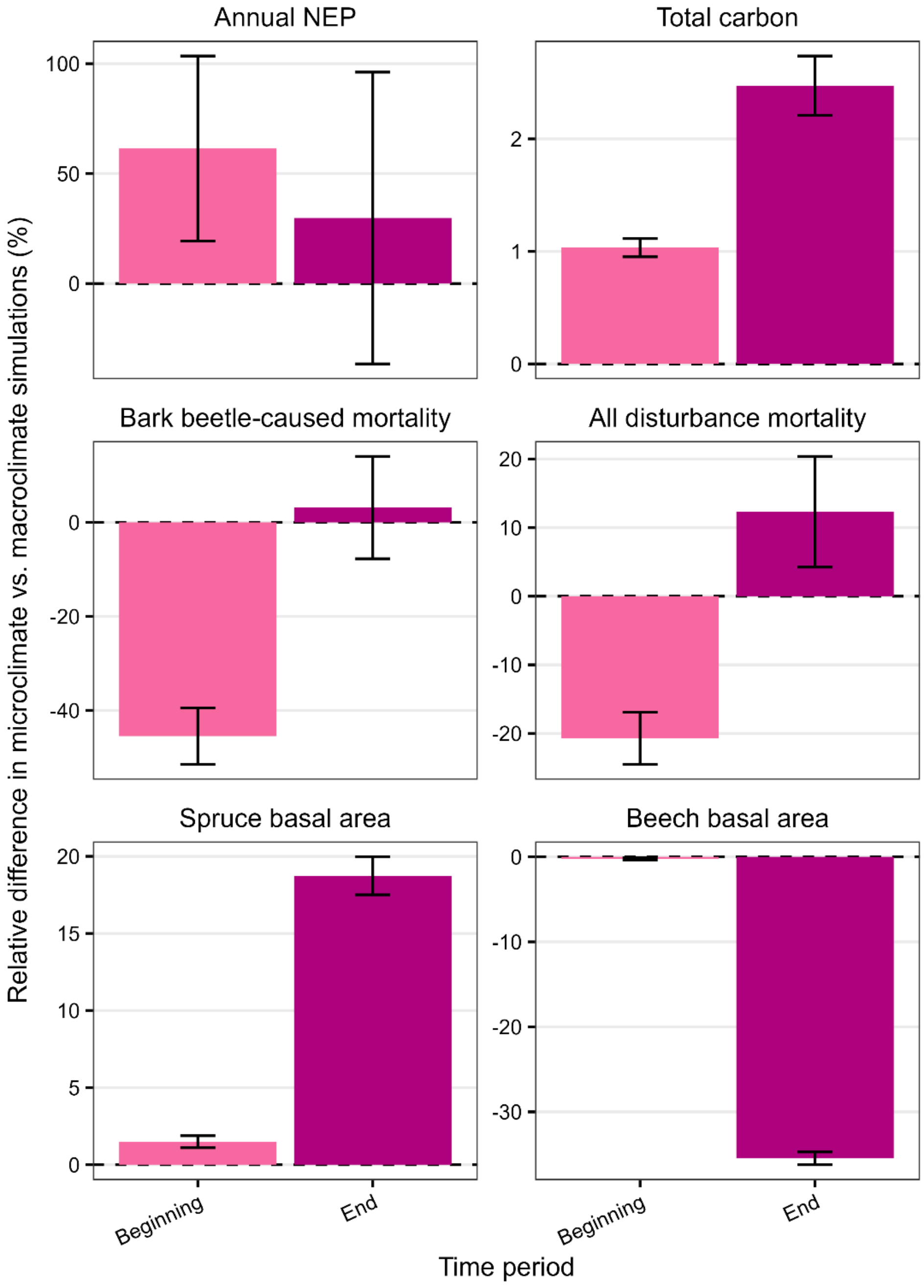

Figure S12. Change in effects of microclimate temperature buffering over time, evaluated by comparing the relative differences in landscape scale indicators at the beginning or end of 1,000 years of forest development. Annual, rather than cumulative, net ecosystem productivity (NEP), bark beetle-caused mortality, and all disturbance mortality were used to ensure comparability across different time periods. All indicators were the average of the first and last 30 simulation years. Relative values were calculated as 100 x (microclimate – macroclimate)/macroclimate. Bar height is the mean value and error bars are two standard errors (n = 10 replicates).

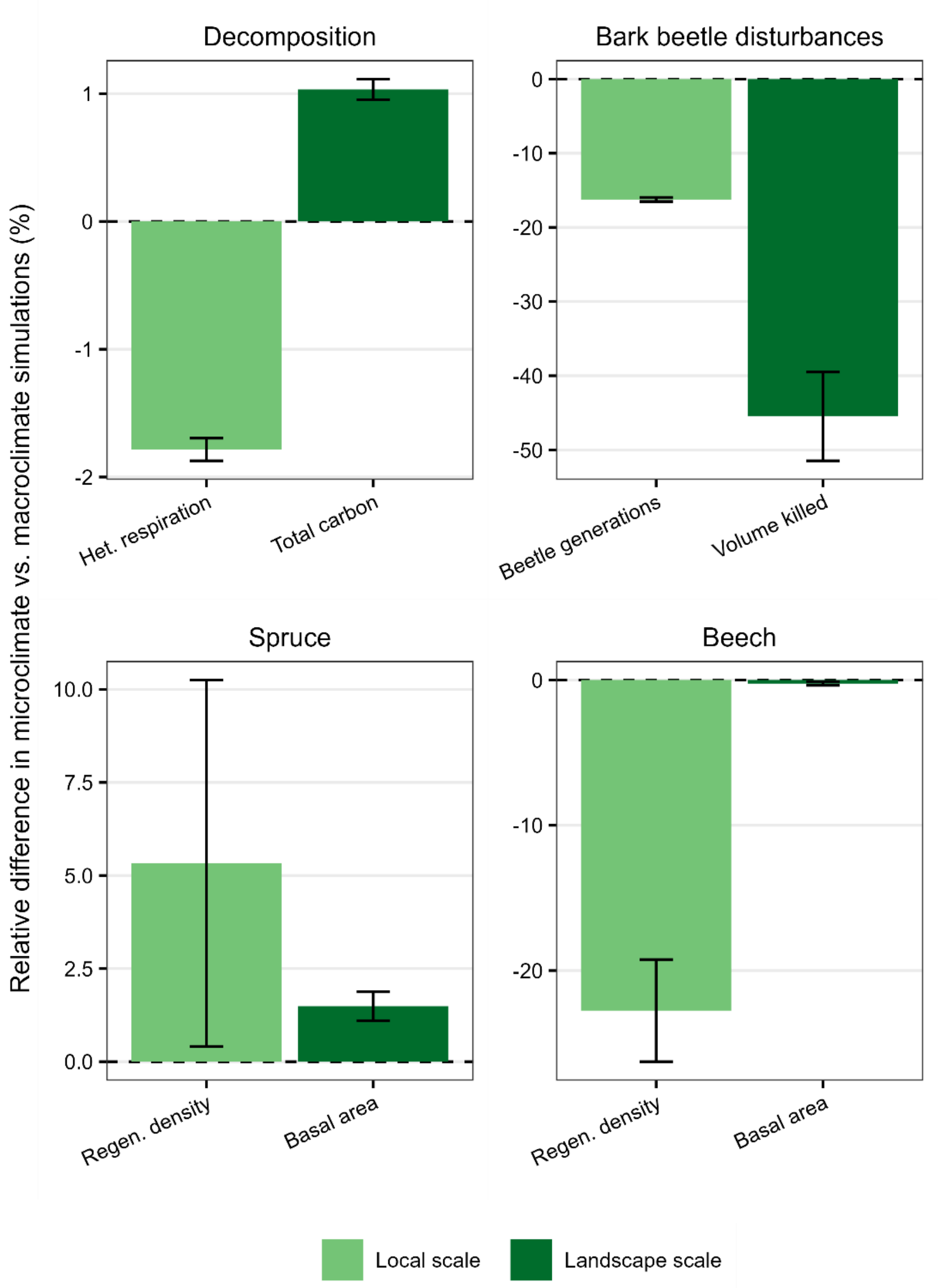

Figure S13. Change in effects of microclimate temperature buffering across scales, evaluated by comparing the relative differences in local and landscape scale indicators, with local scale indicators representing the process directly affected by microclimate temperature. All indicators were the average of the first 30 simulation years. Note that negative relative differences in respiration translate into positive relative differences in carbon (because decreased respiration leads to lower carbon losses to the atmosphere). Relative values were calculated as 100 x (microclimate – macroclimate)/macroclimate. Bar height is the mean value and error bars are two standard errors (n = 10 replicates).
